# The chemical and electrochemical stimuli viologen substituted phthalocyanine with tunable optical features1

**DOI:** 10.55730/1300-0527.3601

**Published:** 2023-05-22

**Authors:** Özge Dilara ATEŞ, Gülenay TUNÇ, Ahmet ŞENOCAK, Burcu DEDEOĞLU, Mehmet Menaf Ayhan, Ayşe Gül Gürek

**Affiliations:** 1 Department of Chemistry, Gebze Technical University, Kocaeli, Turkiye

**Keywords:** Phthalocyanine, charged phthalocyanines, viologen, Zincke reaction, computational analyses

## Abstract

In this study, viologen-tetrasubstituted Zn(II) phthalocyanines (**PcV1** and **PcV2**) were designed and synthesized to achieve the tunable optical features via redox-active viologen groups. Several parameters relevant to the evaluation of the tunable optical features have been investigated: UV-Vis, cyclic voltammetry (CV), EPR, square wave voltammetry (SWV), and theoretical analyses. The results showed that upon reductions and oxidations of viologen groups either chemically or electrochemically, the optical features of **PcV1** and **PcV2** change drastically with switchable processes. These outcomes indicate that achieving control over optical features of large organic chromophores such as Pc with our rational design can be used for the design of new complex organic electronic materials.

## 1. Introduction

Phthalocyanines (Pcs) are one of the most actively studied classes of versatile organic chromophores in the field of fluorescent imaging [1,2], solar cells [3,4], photodynamic therapy [5,6], organic semiconductors [7,8], and batteries [9,10] owing to their convenience of synthesis, excellent photostability, and high delocalized electronic system [11,12]. The photophysical properties (i.e. optical, magnetic, and electrochemical) of Pcs can be altered by incorporation of various groups to the peripheral or nonperipheral positions or metal ions into the core. Moreover, redox-active groups or metals in the Pcs, permit the modulation of these physical properties in a switchable manner by applying different external stimuli (including light, heat, solvents, and presence of various chemical species), which is vital in their technological applicability in such areas [13–15]. Redox-active groups are capable of oxidation and reduction so that electron injection and extraction become possible. There are many types of redox-active groups which usually contain heteroatoms such as nitrogen, oxygen, and sulfur. Among them, the N,N’-disubstituted 4,4-bipyridine, also known as viologen, is widely exploited in numerous materials and compounds where it mostly serves as an electro- and photosensitive active group [16–18]. Viologens are known to undergo chemical or electrochemical one-electron reductions yielding cation radical and neutral state that are stable in the absence of O_2_ [19,20].

However, despite their attractive features, viologens generally suffer from low physicochemical stability. This disadvantage can be suppressed by the incorporation of viologens into large aromatic systems with suitable rigid and symmetric macrocycles such as Pc [21,22]. Particularly, unstable cationic radical species can be stabilized by extended delocalized macromolecules such as Pc or porphyrin. In the meantime, electrochromic feature of viologen can be used to manipulate optical features of Pc. However, viologen attached to Pc by a flexible chain or simply added to Pc solution usually acts as a quencher [14,23–28]. Therefore, in order to transfer the electrochromic feature of viologen to Pc, viologen needs to be incorporated with Pc directly to able to extend conjugation. Otherwise, one-electron reductions of viologen would not affect optical features of Pc.

**Scheme 1:**
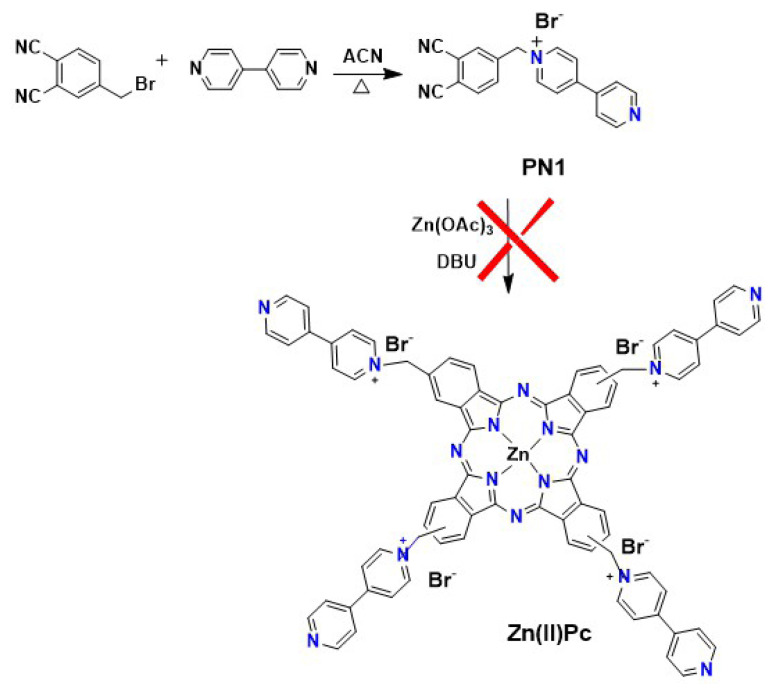
First synthetic route for the synthesis of viologen-linked **Zn(II)Pc** derivative.

**Scheme 2:**
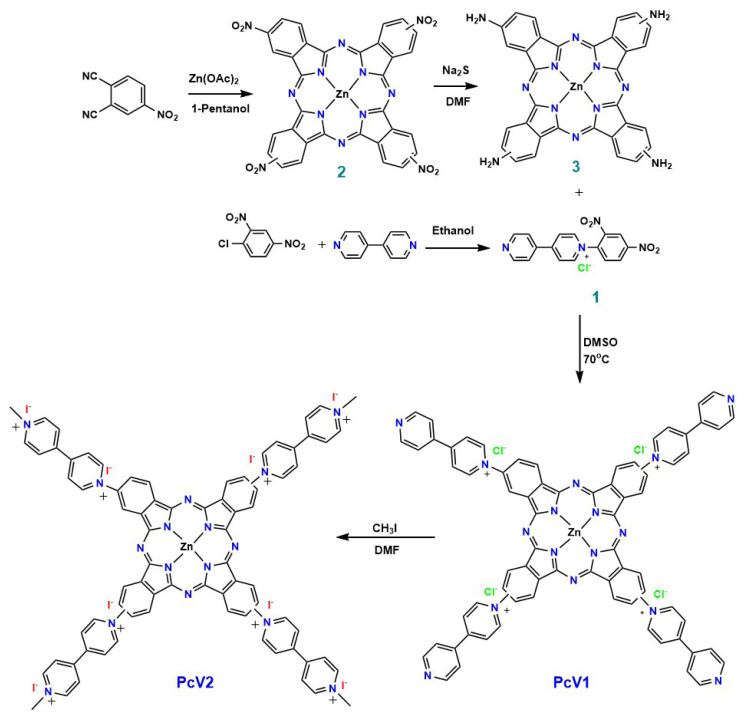
Synthetic route for the preparation of **PcV1** and **PcV2**.

Herein, based on the above thoughts, we report the first, to the best of our knowledge, directly viologen-linked Pc (**PcV1** and **PcV2**) synthesized through the Zincke reaction of ZnPc with viologen Zincke salt, as presented in Schemes 1 and 2. We targeted the chemical and electrochemical oxidation and reduction of the **PcV1** and **PcV2** systems to produce switchable materials, which are responsive to chemical and electrochemical stimuli, with tunable optical features. The structural and spectroscopic changes upon oxidation and reduction in these systems have been studied by UV-Vis, cyclic voltametry (CV), EPR and square wave voltammetry (SWV), and theoretical analyses.

## 2. Experimental

The used equipment, materials, and the electrochemical and theoretical parameters are provided in the SI. The synthesis of **PN1** derivative was performed in accordance with previously reported procedure [29] and its synthesis method was detailed, and spectral data are given in Supplementary Information.

### 2.1. Synthesis and characterization

#### 2.1.1. Synthesis of N-(2,4-dinitrophenyl)-4,4-bipyridinium chloride (1)

4,4′-Bipyridine (2g, 0.013mmol), 2,4-dinitro phenyl chloride (2.6 g, 0.013 mmol) were dissolved in 25 mL anhydrous of EtOH under argon atmosphere stirred at 90 °C for 24 h. After that the reaction mixture was cooled at room temperature and added to 250 mL diethyl ether (Et_2_O), with stirring. The golden-brown solid material obtained was filtered and washed with Et_2_O. Yield: 70%. FT-IR **ν** (cm^−1^) : 3390, 2998, 1639 (C=N), 1609 (C=C),1532, 1458, 1411, 1340 (N-O), 1283, 1237, 1221, 1075, 994, 911, 836, 817, 794, 693. ^1^H NMR (500 MHz, DMSO*-d_6_*) δ ppm : 9.59 (d, 1H), 9.16 (d, 2H), 9.02 (d, 1H) 8.95 (dd, 4H) 8.49 (d, 2H), 8.20 (d, 2H). ^13^C NMR (500 MHz, DMSO*-d_6_*) δ ppm: 155.55, 151.62, 149.67, 147.12, 143.59, 140.90, 138.94, 132.53, 130.72, 125.36, 122.67, 121.92. MALDI-TOF-MS : *m/z* Calcd. for [C_16_H_11_N_4_O_4_Cl] : 358.529; Found 322.531 [M-Cl]^+^.

#### 2.1.2. Synthesis of 2(3),9(10),16(17)23,(24)-tetranitro zinc(II) phthalocyanine (2)

4-Nitro phthalonitrile (900 mg, 5.2 mmol) and Zn(OAc)_2_ (239.6 mg, 1.3 mmol) were dissolved in 1.5 mL anhydrous of DMF under argon atmosphere and stirred. After that, two drops of DBU was added and the reaction mixture was refluxed at 170–180 °C for 24 h. The crude green product was poured into n-hexane (150 mL). The precipitated product was filtered and washed with EtOH (5 × 150 mL). Then, the solid materials was obtained. Yield: 60%. FT-IR **ν** (cm^−1^): 3103 (Ar-CH), 2940–2859, 1640 (C=N), 1611 (C=C) 1522, 1466, 1329 (N-O), 1250, 1184, 1135, 1086, 846, 723. ^1^H NMR (500 MHz, DMSO*-d_6_*) δ ppm: 8.52 (d, 4H), 8.29 (d, 4H), 7.90 (s, 4H). MALDI-TOF-MS: *m/z* Calcd. for [C_32_H_12_N_12_O_8_Zn] : 757.914 ; Found 755.143 [M-3H]^+^, 846.22 [M+3Na+K-3H]^+^, 874.277 [M+5Na+H]^+^, 960.338 [M+DHB+2Na+2H]^+^, 1022,386 [M+DHB+3Na+K+2H]^+^.

#### 2.1.3. Synthesis of 2(3),9(10),16(17)23,(24)-tetraamino zinc(II) phthalocyanine (3)

Compound **2** (700 mg, 0.92 mmol) and Na_2_S (5 g, 0.06 mol) were dissolved in 15 mL anhydrous of dimethylformamide (DMF) under argon atmosphere and stirred at 60 °C for 24 h. During this time period, the reaction was checked by TLC. The reaction mixture was poured into cold water (200 mL) and a greenish solid precipitated. The precipitated product was centrifuged and washed with water and mixtures of MeOH/ Et_2_O (9:1, 50 mL). Then, the solid materials were obtained. Yield: 50%. FT-IR **ν** (cm^−1^): 3328, 3203 (N-H), 3040, 1687, 1605, 1492, 1454, 1405, 1345, 1252, 1134, 1090, 1045, 940, 867, 822, 743. ^1^H NMR (500 MHz, DMSO*-d_6_*) δ ppm: 8.95 (d, 4H), 8.45 (d, 4H), 7.39 (s, 4H), 6.24 (8H, NH). MALDI-TOF-MS : *m/z* Calcd for [C_32_H_20_N_12_Zn]: 637.981; Found 706.090 [M+3Na]^+^, 775.329 [M+6Na ]^+^, 845.203 [M+9Na]^+^.

#### 2.1.4. Synthesis of 2(3),9(10),16(17)23,(24)-tetra-4,4-bipyridine zinc(II) phthalocyanine (PcV1)

Compound **3** (50 mg, 0.078 mmol) and N-(2,4-dinitrophenyl)-4,4-bipyridinium chloride (280 mg, 0.78 mmol) were dissolved in 2 mL dimethylsulfoxide (DMSO) under argon atmosphere and stirred at 70 °C for 24 h. The reaction mixture was poured into n-hexane (10 mL), filtered and washed with n-hexane, DCM, EtOH. Then, the solid materials were obtained. Yield: 55%. FT-IR **ν** (cm^−1^): 3044 (Ar-CH), 1636(C=N), 1610 (C=C), 1543 1488, 1419, 1341, 1218, 1090, 909, 818, 746. ^1^H NMR (500 MHz, DMSO*-d_6_*) δ ppm: 9.54 (d, 8H, ArH), 9.16 (d, 4H, ArH), 8.95 (m, 20H, ArH), 8.44 (m, 4H, ArH), 8.19 (d, 8H, ArH). MALDI-TOF-MS : *m/z* Calcd for [C_72_H_44_N_16_ZnCl_4_] : 1340.458 ; Found 1270.112 [M-2Cl-H]^+^.

#### 2.1.5. Synthesis of 2(3),9(10),16(17)23,(24) tetrametil-4,4-bipyridine zinc(II) phthalocyanine (PcV2)

**PcV1** (50 mg, 0.042 mmol) and CH_3_I (0.5 mL) in 5 mL DMF were stirred at room temperature for 24 h. After pouring into *n*-hexane (25 mL), the resulting precipitate was filtered and washed with acetone and DCM. Then, the green solid material was obtained. Yield: 40. FT-IR **ν** (cm^−1^): 3428, 3030 (Ar-CH), 1634 (C=N), 1608 (C=C), 1531, 1488, 1435, 1339, 1216, 1089, 1067, 910, 818, 741.^1^H NMR (500 MHz, DMSO*-d_6_*) δ ppm**:** 4.56 (m, 12H, CH_3_), 8.93–9.44 (m, 32H, ArH), 10.27–10.74 (m, 12H, ArH). MALDI-TOF-MS : *m/z* Calcd for [C_76_H_56_N_16_ZnI_8_]: 2273.986 ; Found 1258,867 [M-8I]^+^, 1297.213 [M-8I+K]^+^.

## 3. Results and discussion

### 3.1. Synthesis and characterization of phthalocyanines

The aim of this study is to synthesize viologen-substituted new symmetric zinc Pc derivatives. As a general strategy, viologens can be synthesized from their precursor 4,4′-bipyridine via N-alkylation with suitable alkyl groups or 1-chloro-2,4-dinitrobenzene to yield the corresponding N-alkylated salt or Zincke salt. Thus, N-alkylation and Zincke reactions are extensively used synthetic approaches to prepare simple viologens. From a molecular design point of view, two different synthetic routes were used for the synthesis of viologen-linked Zn(II)Pc derivatives (Schemes 1 and 2). The first synthetic strategy envisaged was the preparation of a symmetric phthalocyanine on which the viologen function is introduced (Scheme 1). In this method, a viologen-substituted new phthalonitrile (**PN1**) was used to obtain Zn(II)Pc, but the purification and separation of the desired Zn(II)Pc from the reaction mixture proved to be impossible. For the preparation of viologen-linked Pc derivatives **PcV1** and **PcV2**, we adopted the alternative synthetic strategy depicted in Scheme 2.

The synthesis procedures of tetra-nitro (**2**) and tetra-amino (**3**) Pc derivatives have been modified and recharacterized by using different spectroscopic techniques (Figures S1–S9). The viologen-linked Pc derivative **PcV1** was prepared through the Zincke reaction between tetra-amino Zn(II)Pc and N-(2,4-dinitrophenyl)-4,4-bipyridinium chloride in DMSO at 70 °C under argon atmosphere for 24 h. The quaternization of **PcV1** was achieved by using methyl iodide in DMSO to obtain **PcV2**.

^1^H- and ^13^C- NMR, FT-IR, and MALDI-TOF MS techniques were used to verify the proposed structures (see supplementary section, Figures S1–S19). In the MALDI-TOF mass spectra of **PcV1** and **PcV2**, peaks were observed for **PcV1** at m/z 1201.288 [M-4Cl-3H]^+^, 1254.647 [M-2Cl-H]^+^, and 1271.219 [M+Na-2Cl+4H]^+^; for **PcV2**, at m/z 1258.867 [M-8I]^+^, 1297.213 [M-8I+K]^+^.

^1^H-NMR spectra of **PcV1** and **PcV2**, were achieved in DMSO-*d_6_*. The quaternization of **PcV1** can be conveniently monitored by ^1^H-NMR spectroscopy. The most characteristic change in the NMR spectrum of **PV2** is the appearance of the peak belonging to the methyl group at 4.5 ppm. All these results are compatible with the defined structures.

### 3.2. Analysis of photophysical properties

**Figure 1:**
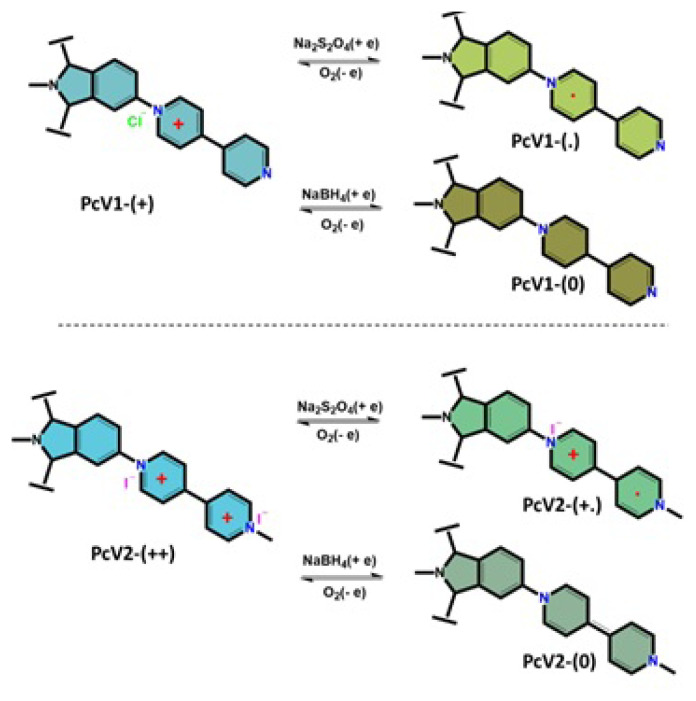
The chemical or electrochemical one-electron reductions of **PcV1** and **PcV2**.

The photophysical properties of **PcV1**, **PcV2** and their oxidation states in solution at 1 × 10^−5^ M were investigated by UV-Vis spectrophotometry. To investigate the redox properties of **PcV1** and **PcV2**, sodium dithionite (Na_2_S_2_O_4_) was used to obtain radical forms (**PcV1-(.)** and **PcV2-(+.)**) and sodium borohydride (NaBH_4_) was used to obtain neutral forms (**PcV1-(0)** and **PcV2-(0)**) (Figure 1).

**Figure 2:**
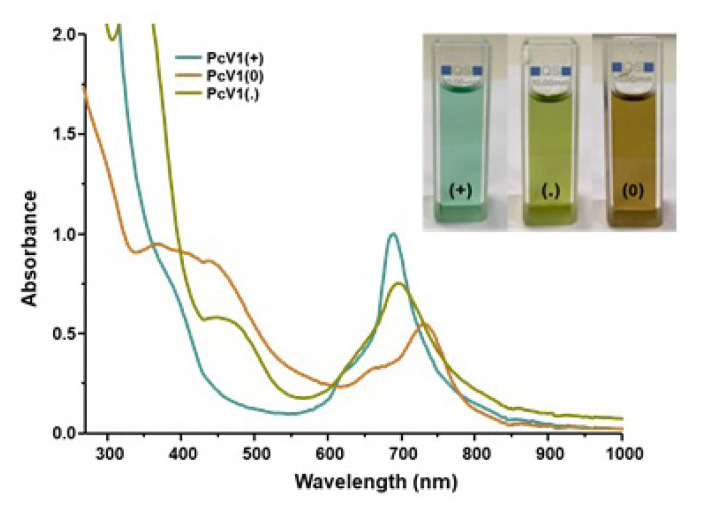
The absorption spectra of **PcV1-(+)**, **PcV1-(.)**, and **PcV1-(0)** in DMSO (1 × 10^−5^ M).

**Figure 3:**
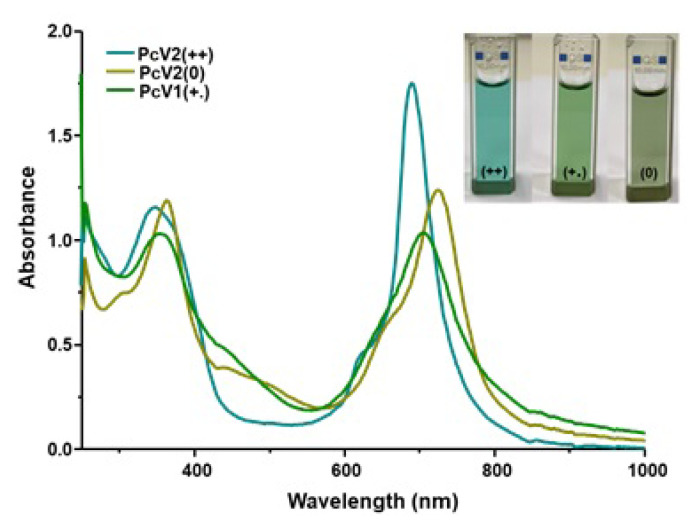
The absorption spectra of **PcV2-(++)**, **PcV2-(+.)**, and **PcV2-(0)** in DMSO (1 × 10^−5^ M)

In this study, the electronic spectra of **PcV1-(+)** and **PcV2-(++)** displayed characteristic absorption in the Q band region at around 688 and 690 nm, respectively. Addition of Na_2_S_2_O_4_ to the solution of the **PcV1-(+)** and **PcV2-(++)**, the color of solutions immediately changed from green to green-yellow for **PcV1-(+)** and blue to green for **PcV2-(++)** (Figures 2 and 3), indicating the formation of a radical by the single-electron reduction of the viologen unit. When the radical solution was exposed to air, the green-yellow and green color gradually changed back to green and blue by aerobic oxidation, indicating the occurrence of reversible reduction and oxidation processes. Furthermore, the absorption band of both **PcV1-(.)** and **PcV2-(+.)** red shifted due to the formation radical induced extension of delocalization (Figures 2 and 3).

The addition of NaBH_4_ to **PcV1-(+)** and **PcV2-(++)** solutions leads to the formation of neutral **PcV1-(0)** and **PcV2-(0)** which change color from green to brown for **PcV1-(+)** and blue to green for **PcV2-(++)** (Figures 2 and 3). Similarly, neutral **PcV1-(0)** and **PcV2-(0)** solution have reversible reduction and oxidation processes by aerobic oxidation. As anticipated, the absorption bands of neutral forms are red shifted due to large extended delocalization and partial aggregation.

### 3.3. Electron paramagnetic resonance (EPR)

**Figure 4:**
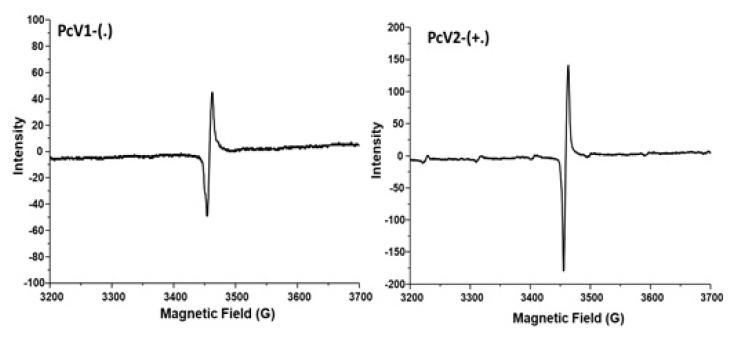
Electron spin resonance spectra for **PcV1-(.)** and **PcV2-(+.)**.

To further confirm the generation of the radicals of the **PcV1-(.)** and **PcV2-(+.)**, the electron paramagnetic resonance (EPR) spectra (Figure 4) were measured after addition of Na_2_S_2_O_4_ to the solution.

**Figure 5:**
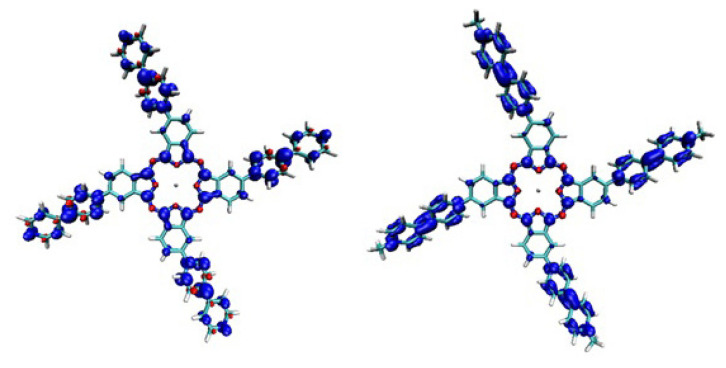
Spin-density isosurfaces (at ±0.002 au, blue – positive density, green – negative density) of **PcV1-(.)** and **PcV2-(+.)** radicals.

There are no EPR signals before the addition of Na_2_S_2_O_4_, but after the addition of Na_2_S_2_O_4_, a characteristics of viologen radical single line signal appears indicating the formation of **PcV1-(.)** and **PcV2-(+.)**. This demonstrates that **PcV1-(+)** and **PcV2-(++)** cations are reduced to **PcV1-(.)** and **PcV2-(+.)** radicals, suggesting the electron transfer occurs. Spin density plots (Figure 5) show a strong delocalization of positive (blue) spin density onto the viologen group of both **PcV1-(+)** and **PcV2-(++)** which agrees with the results of the EPR spectra.

### 3.4. Optical band gap measurements

**Figure 6:**
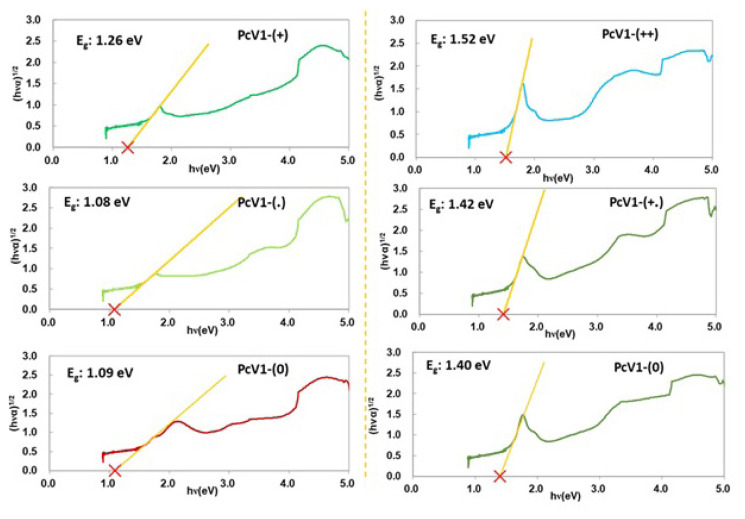
The indirect bandgap measurement of three redox states of **PcV1** and **PcV2** via Tauc plotting of the transmittance spectra.

The optical band gaps of three oxidation states of **PcV1** and **PcV2** in solution have been investigated with reflectance spectroscopy (Figures S17 and S18) using the relational expression proposed by Tauc [30,31], Davis, and Mott [32] and calculation details are given in Supplementary Information. The reflectance spectra and Tauc plots for three redox states of **PcV1** and **PcV2** are shown in Figure 6.

The Tauc plot derived from first jump in the spectrum yields a narrow optical band gap at 1.26 and 1.52 eV for **PcV1-(+)** and **PcV2-(++)** cations, respectively. The optical band gap of **PcV1-(.)** (1.08 eV) and **PcV2-(+.)** (1.42 eV) decreases considerably compared to cation states, whereas band gap of **PcV1-**(0) (1.09 eV) and **PcV2**-(0) (1.40 eV) either slightly increases or decreases. This tunability of the optical band gap of Pc derivatives is mainly attributed to the extension level of conjugation via reduction and oxidation of viologen group. Our data displays that band gaps of the **PcV1** and **PcV2** derivatives can be tuned in the ranges of 1.26–1.09 and 1.52–1.40 eV by controlling redox states.

**Figure 7:**
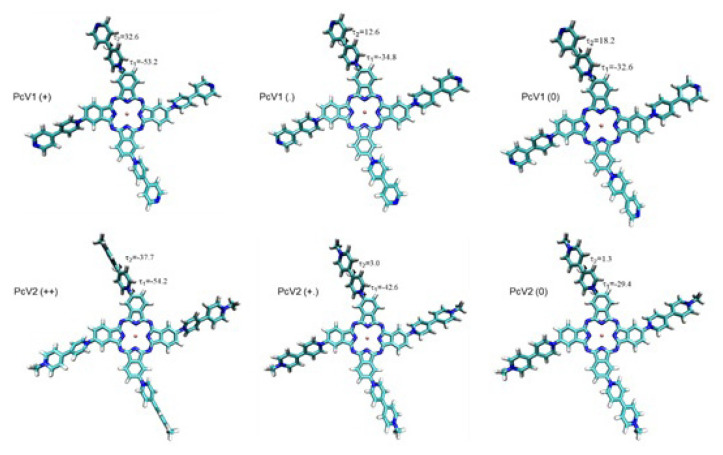
Optimized structures of cationic, radical, and neutral states of **PcV1** and **PcV2** with the selected dihedral angles shown on the three-dimensional molecular images.

Furthermore, optimized geometries of cationic, radical, and neutral states of **PcV1** and **PcV2** are depicted in Figure 7 with the selected dihedral angles. τ_1_ represents the position of the viologen substituent with respect to the phthalocyanine plane, while τ_2_ indicates the distortion of two constituent rings of the viologen itself. Both τ_1_ and τ_2_ deviates from planarity in the cationic form of **PcV1** and **PcV2**, and both reduce significantly in the radical and neutral forms enhancing the delocalization.

### 3.5. The electrochemical characterization

The rich redox activity of the Pc ring improved the electrochemical and electrochromic performances of viologen substituents. The electrochemical characterizations of **PcV1** and **PcV2** were evaluated in DMSO/TBAP electrolyte by CV and SWV techniques. The voltametric responses of each compound were different due to formations of oxidized and reduced radical complexes. Table presents the electrochemical parameters of **PcV1** and **PcV2** such as the first oxidation and first reduction half-wave peak potentials (E_1/2_), HOMO and LUMO energies which were determined by using CV responses and band gap energies were estimated from both voltametric responses (Eg^ec^) and UV-Vis measurements as Eg^opt^ (Table).

**Figure 8:**
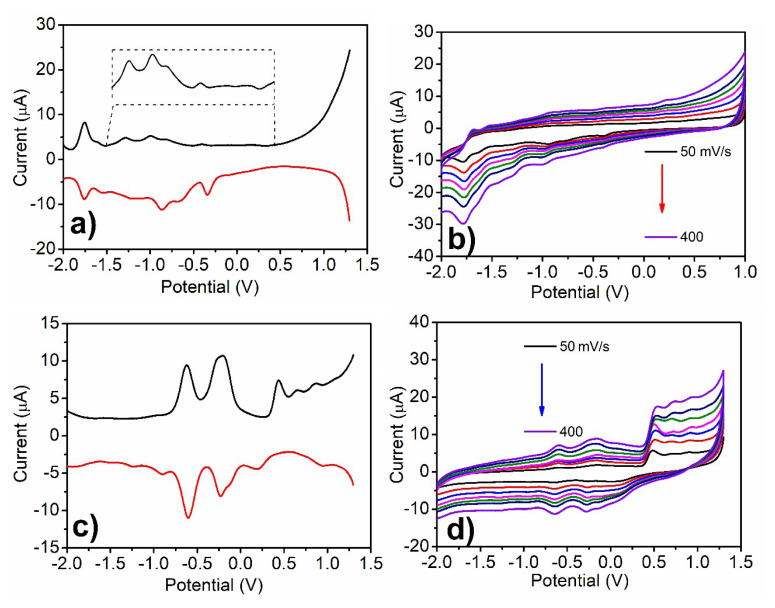
a) SWVs and b) CVs at various scan rates of **PcV1**, c) SWVs, and d) CVs at various scan rates of **PcV2** on GCE in DMSO/TBAP.

The electrochemical measurements for **PcV1** are depicted in Figures 8a and 8b. ΔE_p_ values of the reduction couples obtained by increasing scan rates from 50 to 400 mV/s. The proportion of the anodic peak current and cathodic peak current of these couples were similar for all scan rates which indicates reversibility of the redox process [33]. **PcV1** has one oxidation peak which is irreversible process at 0.20 V and five reduction waves at −0.42, −0.65, −0.98, −1.29, and −1.77 V. Only one of them (−1.77 V) is reversible, while the others are irreversible. Some of the redox peaks of **PcV1** were not clearly visible from the CV, but more clear results were obtained from the SWV measurements. Pc ring-based redox processes were clearly observed at 0.20 V, −0.98, 1.29, and −1.77 V and the others at −0.42 and −0.65 V belonged to viologen substituent-based reduction waves. In addition, SWV and CV results of **PcV2** are demonstrated in Figures 8c and 8d. Contrary to the **PcV1**, **PcV2** has more prominent peaks which are located at −1.22, −0.89, −0.60, −0.25, 0.45, 0.65, and 0.87 V. The reduction peak at −0.60 and −0.25 V were reversible and quasireversible, respectively, which belonged to viologen substituent-based reduction. **PcV2** has high reduction and oxidation activities, the main reason for which is that it contains methyl groups, which causes an increase in electrochemical activity and therefore in electrochromic properties. Moreover, the other peaks have irreversible characteristics, which are observed by both CV and SWV measurements. The reductions were expected to be observed at more positive potentials for **PcV2** due to the smaller electronegativity of iodine than chlorine anion in the complex system. There are a few studies on asymmetric Pc derivatives containing viologen groups in the literature [14, 23, 34] and these redox behaviors are accompanied with the viologen and methyl-viologen appended asymmetric ZnPc study reported by Şener et al. They reported that the cation radicals can be obtained by reduction of dicationic viologen as reversible; however, neutral viologen can be obtained by electrochemical the reduction of the cation radicals as irreversible [14]. Therefore, **PcV2** has more irreversible oxidation and reduction states than those of **PcV1** due to its dicationic nature and methyl viologen substituent. The redox processes of Pc core were clearly observed at −1.22, −0.89, 0.45, 0.65, and 0.87 V for **PcV2**. The peak currents for the oxidation and reduction couples of the **PcV1** and **PcV2** were usually found to be directly proportional to the square root of scan rate, indicating their diffusion-controlled processes. The optical changes were observed for **PcV1** and **PcV2** macrocycles during the electrochemical processes in DMSO/TBAP electrolyte solutions. The decrease in Q band at 685 nm was monitored without shifting in the visible region of absorption spectra for **PcV1** and **PcV2** macrocycles for oxidation processes with applying 1.5 V (Figures S19a and S19b). On the other hand, new broad absorption bands appeared within the range of 400–600 nm during the reduction processes with applying −2.0 V (Figures S19c and S19d). The results obtained from spectroelectrochemical processes supported the optical changes during the chemical oxidation and reduction with NaBH_4_ and Na_2_S_2_O_4_, which were characteristic for ring-based electrochemical behavior in Pc compounds [34]. The obtained optical results support the ligand-based electrochemical behavior of these Pc compounds.

## 4. Conclusion

In this study, we have synthesized directly viologen-linked **PcV1** and **PcV2** to extend the tunable optical features of the Pc ring with electrochromic viologen derivatives. We have investigated the chemical and electrochemical oxidation and reduction of the **PcV1** and **PcV2** systems by using UV-Vis, cyclic (CV) and square wave voltammetry (SWV), and theoretical analyses. Upon formation of radical of the **PcV1-(.)** and **PcV2-(+.)**, with the addition of Na_2_S_2_O_4_ to the solution of the **PcV1-(+)** and **PcV2-(++)**, or formation of the neutral **PcV1-(0)** and **PcV2-(0)**, with the addition of NaBH_4_ to **PcV1-(+)** and **PcV2-(++)** solutions, the optical features were drastically altered with reversible reduction and oxidation processes. Furthermore, theoretical analyses showed that formation of radical and neutral forms of **PcV1** and **PcV2** led to not only the significantly red shifted absorption bands but also reduce dihedral angles of viologen group deviation from planarity. These results indicate that the incorporation of viologen to Pc can be a useful and efficient tool to adjust the optical features of Pc complexes with switchable processes. Hence, we believe that this systematic study to control over optical features of Pc complexes will prove to be of key importance for the future rational design of complex organic electrochromic materials in various applications such as sensor devices, and organic semiconductors.

## Supplementary Information

### Materials and methods

All reagents, purchased from fine chemical suppliers Aldrich, Merck, Alfa Aesar, and Fluka, were used without further purification unless otherwise stated. Solvents were either used as commercially supplied or used as purified by standard techniques. Infrared spectra were recorded between 4000 and 650 cm^−1^ using a PerkinElmer FT-IR System Spectrum BX spectrometer with an attenuated total reflection (ATR) accessory featuring a zinc selenide (ZnSe) crystal. ^1^H NMR spectra were recorded on Bruker and Varian INNOVA 500 MHz spectrometers. MALDI-TOF-MS measurements were performed on a Bruker Daltonics MicrOTOF spectrometer. Positive ion and linear mode MALDI-TOF-MS spectra of the compounds were obtained in 2,5-dihydroxy benzoic acid (DHB) or dithranol (DIT) MALDI matrixes using nitrogen laser accumulating 50 laser shots. The absorption spectra of the dyes and the sensitized films were measured using Shimadzu UV 2600 spectrophotometer. Electron paramagnetic resonance (EPR) signals were recorded using Bruker EMX X-band spectrometer (9.8 GHz).

### Electrochemical analyses

Electrochemical identifications were carried out with cyclic voltammetry (CV) and square wave voltammetry (SWV) measurements on CH Instruments 440B model workstation. The setup was conventional three-electrode cell equipped with glassy carbon working electrode, platinum wire counter electrode, and Ag/AgCl reference electrode. All measurements of samples were recorded as reported analyte concentration in 0.1 M Bu_4_NPF_6_ electrolyte solutions of dimethylsulfoxide. High purity of nitrogen gas was used to deoxygenate the solution at least 10 min prior to each run and maintain a nitrogen blanket. All electrochemical measurements were performed at ambient temperature, at scanning rates of 400 mV/s, 300 mV/s, 250 mV/s, 200 mV/s, 150 mV/s, 100 mV/s, and 50 mV/s, respectively. Ferrocene was used as internal reference and all potentials were referenced to ferrocene/ferrocenium (Fc/Fc+) redox couple. In situ spectroelectrochemical characterizations of synthesized Pc molecules were performed by using a quartz thin-layer spectroelectrochemical cell at 25 °C including a three-electrode configuration as the reference, counter and working electrodes were a Ag/AgCl, Pt wire and a Pt gauze semitransparent. The highest occupied molecular orbital (HOMO) and the lowest unoccupied molecular orbital (LUMO) were calculated from E_HOMO_ = – [(E_ox_^onset^ – E_1/2(ferrosen_) + 4.8] and E_LUMO_ = – [(E_red_^onset^_–_ E_1/2(ferrosen_) + 4.8] equations values. The band gap can be obtained from the UV-vis absorption band edge (λ_onset_) using equation Eg = 1242 / λ_onset_ [S1].

### Computational analyses

Geometry optimizations were performed using the density functional B3LYP as implemented in Gaussian16 [S2]. For zinc LANL2DZ effective core potential, and for the rest of the atoms 6–31G(d) basis sets were used. The unrestricted open-shell formalism was employed for radical species. Frequency analyses were performed on the optimized geometries to confirm the nature of the stationary points. The effect of a polar environment has been considered by the integral equation formalism polarizable continuum model (IEF-PCM), with dimethyl sulfoxide (ɛ = 46.83) as the solvent. [S3–S5] Spin densities were computed at the same level of theory and plotted to analyze the extent of delocalization of the unpaired electron within the molecular framework.

The tetrasubstituted phthalocyanines are known to have four constitutional isomers of D_2h_, C_4h_, C_2v_, and Cs. The isomer distribution of the product depends on the reaction conditions. Among these isomers, C_4h_ isomer has even electron distribution and therefore has been chosen for our theoretical calculations.

### Synthesis of 1-(3,4-dicyanobenzyl)-[4,4′-bipyridine]-1-ium (PN1)

4,4′-bipyridine (2.75 g, 0.018mmol) was dissolved in acetonitrile (ACN) (40 mL) under argon atmosphere stirred at 80 °C for 1 h. 4-bromomethyl phthalonitrile (500 mg, 2.26 mmol) was dissolved in 40 mL and then it was added to reaction mixture. After that the reaction mixture was stirred at 80 °C for 24 h, and it was cooled to room temperature. The light yellow crystalize compound was obtained and added to 250 mL diethyl ether (Et_2_O), with stirring. The golden-brown solid material was filtered and washed with DCM. Yield: 88%. FT-IR **ν** (cm^−1^): 3111 (Ar-CH), 3108–2980 (CH_2_), 2238 (CºN), 1340 (N-O), 1639 (−C=N−), 1595, 1543, 1523,1466, 1355, 1294, 1226, 1171, 992, 920, 886, 815, 776, 724. ^1^H NMR (500 MHz, DMSO*-d_6_*) δ ppm: 8.95 (d, 2H, ArH), 8.84 (d, 2H, ArH), 8.75 (d, 2H, ArH), 8.16 (d, 1H, ArH), 8.13 (s, 1H, ArH), 7.69 (d, 1H, ArH), 5.96 (s, 2H, CH_2_), C_19_H_13_N_4_Br (M_w_= 377) MALDI-TOF-MS(found) m/z: 296.112 [M-Br]^+^.

Figure S1 FT-IR spectrum of 1-(3,4-dicyanobenzyl)-[4,4′-bipyridine]-1-ium (**PN1**).

Figure S2 MS (MALDI-TOF) spectrum of 1-(3,4-dicyanobenzyl)-[4,4′-bipyridine]-1-ium (**PN1)** (matrix: DHB).

Figure S3 ^1^H-NMR spectrum of 1-(3,4-dicyanobenzyl)-[4,4′-bipyridine]-1-ium (**PN1**) (in DMSO -*d_6_*).

Figure S4 FT-IR spectrum of tetra-nitro phthalocyaninato zinc(II) (**2**).

Figure S5 MS (MALDI-TOF) spectrum of tetra-nitro phthalocyaninato zinc(II) (**2**) (matrix: DHB).

Figure S6 ^1^H-NMR spectrum of tetra-nitro phthalocyaninato zinc(II) (**2**) (in DMSO *-d_6_*).

Figure S7 FT-IR spectrum of tetra-amino phthalocyaninato zinc(II) (**3**).

Figure S8 MS (MALDI-TOF) spectrum of tetra-amino phthalocyaninato zinc(II) (**3**) (matrix: DHB).

Figure S9 ^1^H-NMR spectrum of tetraamino phthalocyaninato zinc(II) (**3**) (in DMSO *-d_6_*).

Figure S10 FT-IR spectrum of N-(2,4-dinitrophenyl)-4,4-bipyridinium chloride (**1**).

Figure S11 MS (MALDI-TOF) spectrum of N-(2,4-Dinitrophenyl)-4,4-bipyridinium chloride (**1**).

Figure S12 ^1^H-NMR spectrum of N-(2,4-Dinitrophenyl)-4,4-bipyridinium chloride (**1**) (in DMSO *-d_6_*).

Figure S13 ^13^C-NMR spectrum of N-(2,4-Dinitrophenyl)-4,4-bipyridinium chloride (**1**) (in DMSO *-d_6_*).

Figure S14 FT-IR spectrum of compound **PcV1**.

Figure S15 MS (MALDI-TOF) spectrum of compound **PcV1**.

Figure S16 ^1^H-NMR spectrum of compound **PcV1** (in DMSO *-d*

Figure S17 FT-IR spectrum of compound **PcV2**.

Figure S18 MS (MALDI-TOF) spectrum of compound **PcV2**.

Figure S19 ^1^H-NMR spectrum of compound **PcV2** (in DMSO *-d_6_*)

### Band gap measurement

The optical band gaps of three oxidation states of **PcV1** and **PcV2** have been investigated with evaluation of transmittance with the following relational expression (1) proposed by Tauc, Davis, [S6,S7] and Mott [S8] is used. Data for these plots were obtained from the transmittance versus wavelength spectral data run using Shimadzu 2600 UV-Vis-NIR spectrometer.


(1) $${\left(h\nu \alpha \right)}^{1/n}=A(h\nu -\mathrm{Eg})$$

Here: **h:** Planck’s constant, **ν:** frequency of vibration, **α:** absorption coefficient, **Eg:** band gap, **A:** proportional constant. The value of the exponent **n** denotes the nature of the sample transition. For direct allowed transition **n = 0.5** and for indirect allowed transition **n = 2**. The band gap values were determined from the intersect of the tangent of the onset of reflection, expressed as (hνα)^1/n^ with the x-axis. We found that the direct allowed transition Tauc factor (n = 0.5) cannot provide a satisfactory fitting. However, a decent linear fit was obtained for n = 2, which indicates that band gaps of three oxidation state of **PcV1** and **PcV2** possess allowed indirect transitions. Therefore, the indirect allowed transition Tauc factor **n = 2** is used for all our calculations.

Figure S17 Transmittance spectra of **PcV1**-(+), **PcV1**-(.) and **PcV1**-(0) in DMSO (1 × 10-−^5^ M).

Figure S18 Transmittance spectra of **PcV2**-(++), **PcV2**-(+.) and **PcV2**-(0) in DMSO (1 × 10-^−5^ M).

Figure S19 In situ UV-vis spectral changes during the electrolysis of **PcV1** (a and c) and **PcV2** (b and d) at various constant potentials in DMSO /TBAP electrolyte system.

ReferencesS1
LeonatL
SbârceaG
BrânzoiIV
Cyclic voltammetry for energy level estimation of organic materialsUPB Scientific Bulletin, Series B: Chemistry and Materials Science2013751111181454-2331S2Gaussian 16, Revision A03
FrischMJ
TrucksGW
SchlegelHB
ScuseriaGE
RobbMA
CheesemanJR
ScalmaniG
BaroneV
PeterssonGA
NakatsujiH
LiX
CaricatOM
MarenichA
BloinoJ
JaneskoBG
GompertsR
MennucciB
HratchianHP
OrtizJV
IzmaylovAF
SonnenbergJL
Williams-YoungD
DingF
LippariniF
EgidiF
GoingsJ
PengB
PetroneA
HendersonT
RanasingheD
ZakrzewskiVG
GaoJ
RegaN
ZhengG
LiangW
HadaM
EharaM
ToyotaK
FukudaR
HasegawaJ
IshidaM
NakajimaT
HondaY
KitaoO
NakaiH
VrevenT
ThrossellK
MontgomeryJA
PeraltaJE
OgliaroF
BearparkM
HeydJJ
BrothersE
KudinKN
StaroverovVN
KeithT
KobayashiR
NormandJ
RaghavachariK
RendellA
BurantJC
IyengarSS
TomasiJ
CossiM
MillamJM
KleneM
AdamoC
CammiR
OchterskiJW
MartinRL
MorokumaK
FarkasO
ForesmanJB
FoxDJ
Gaussian, IncWallingford CT2016S3
CancèsE
MennucciB
TomasiJ
A new integral equation formalism for the polarizable continuum model: Theoretical background and applications to isotropic and anisotropic dielectricsThe Journal of Chemical Physics19971073032304110.1063/1.474659S4
MennucciB
CancèsE
TomasiJ
Evaluation of solvent effects in isotropic and anisotropic dielectrics and in ionic solutions with a unified integral equation method: theoretical bases, computational implementation, and numerical applicationsJournal of Physical Chemistry B1997101105061051710.1021/jp971959kS5
MennucciB
TomasiJ
Continuum solvation models: A new approach to the problem of solute’s charge distribution and cavity boundariesThe Journal of Chemical Physics19971065151515810.1063/1.473558S6
TaucJ
GrigoroviciR
VancuA
Optical properties and electronic structure of amorphous germaniumBasic solid state physic19661562563710.1002/pssb.19660150224S7
TaucJ
Optical Properties of SolidsAmsterdam, North-Holland Pub Co; New YorkAmerican Elsevier1972S8
MottNF
DavisEA
Conduction in non-crystalline systems V Conductivity, optical absorption and photoconductivity in amorphous semiconductorsThe Philosophical Magazine: A Journal of Theoretical Experimental and Applied Physics19702290392210.1080/14786437008221061

## Figures and Tables

**Table:** HOMO and LUMO energy level values determined from experimental cyclic voltammetry (CV) and band gap energy values found as a result of UV-vis measurements.

Compound	E_red_^onset^ (V)	E_LUMO_ (eV)	E_ox_^onset^ (V)	E_HOMO_ (eV)	Eg^ec^ (eV)	Eg^opt^ (eV)
**PcV1**	−0.42	−3.98	0.20	−4.60	0.62	**1.49**
**PcV2**	−0.25	−4.15	0.45	−4.85	0.70	**1.48**

Eg^ec^ : Electrochemical band gap, Eg^opt^: Optical band gap.
