# Supplementary figures and images for: The chemical and electrochemical stimuli viologen substituted phthalocyanine with tunable optical features1
Source: Turk J Chem. 2023 May 22;47(5):1149–59. doi: 10.55730/1300-0527.3601 (PMC10760869; doi:10.55730/1300-0527.3601)

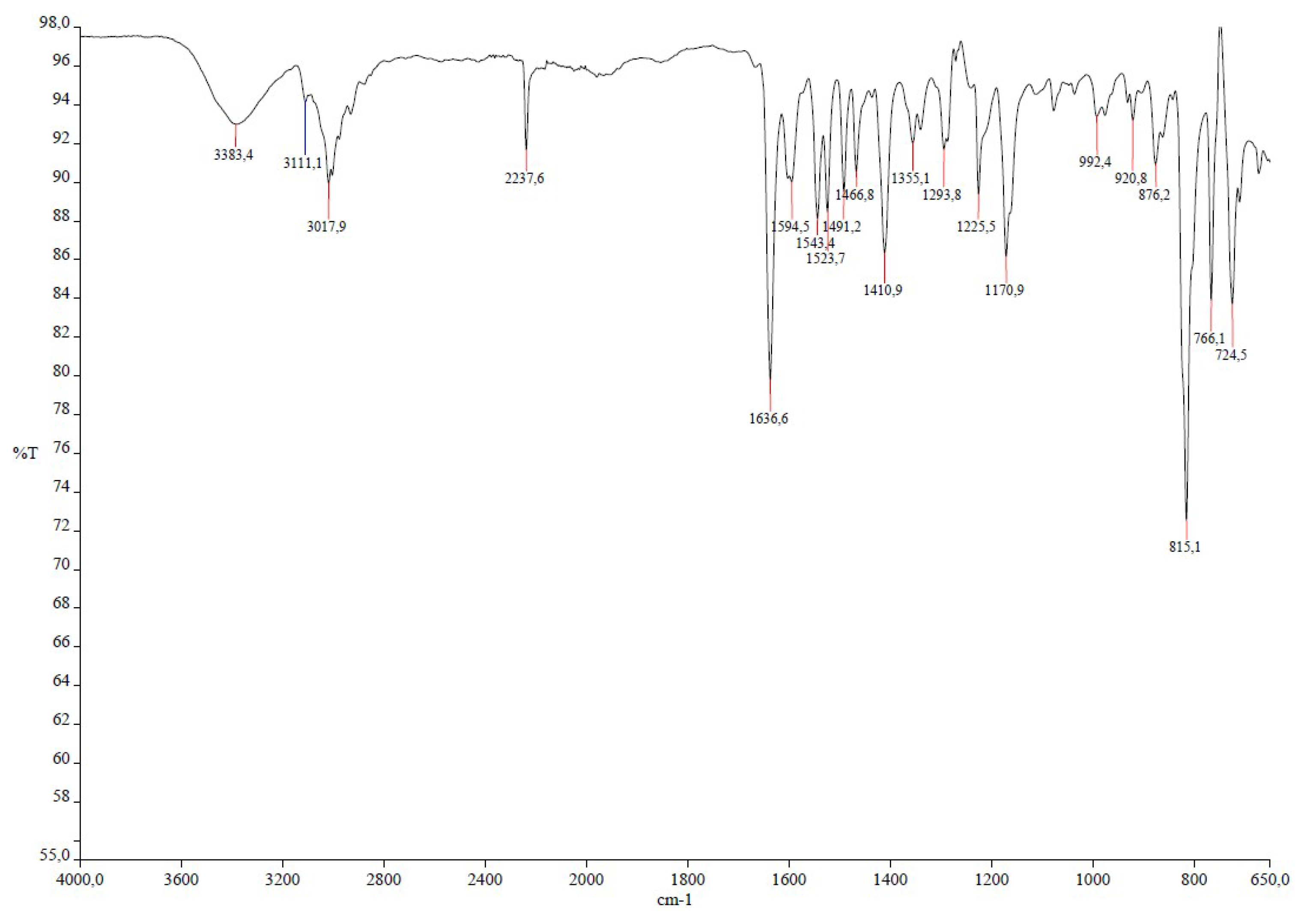

Supplement: Figure S1 — FT-IR spectrum of 1-(3,4-dicyanobenzyl)-[4,4′-bipyridine]-1-ium (PN1). [file turkjchem-47-5-1149s1.tif]

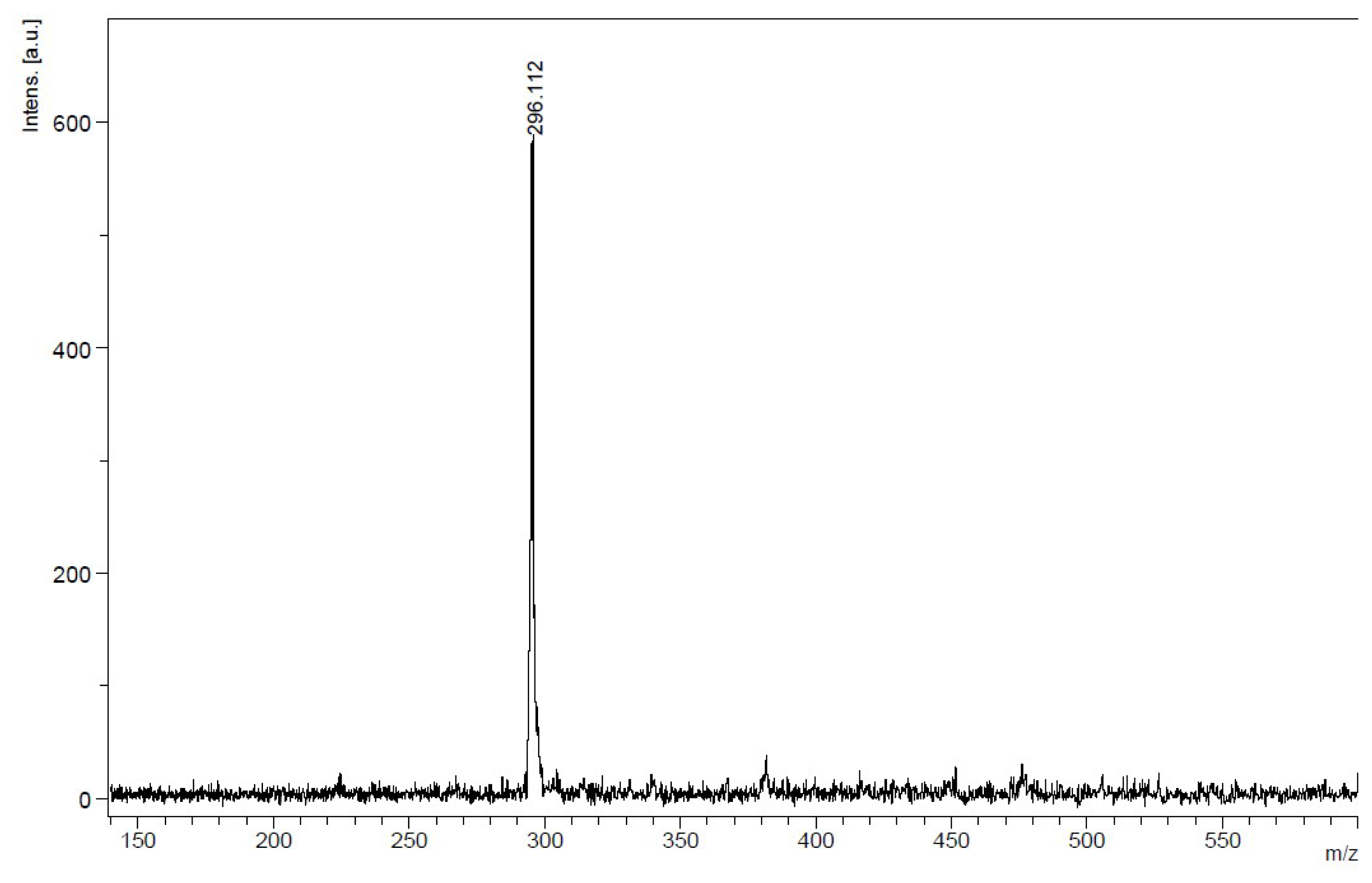

Supplement: Figure S2 — MS (MALDI-TOF) spectrum of 1-(3,4-dicyanobenzyl)-[4,4′-bipyridine]-1-ium (PN1) (matrix: DHB). [file turkjchem-47-5-1149s2.tif]

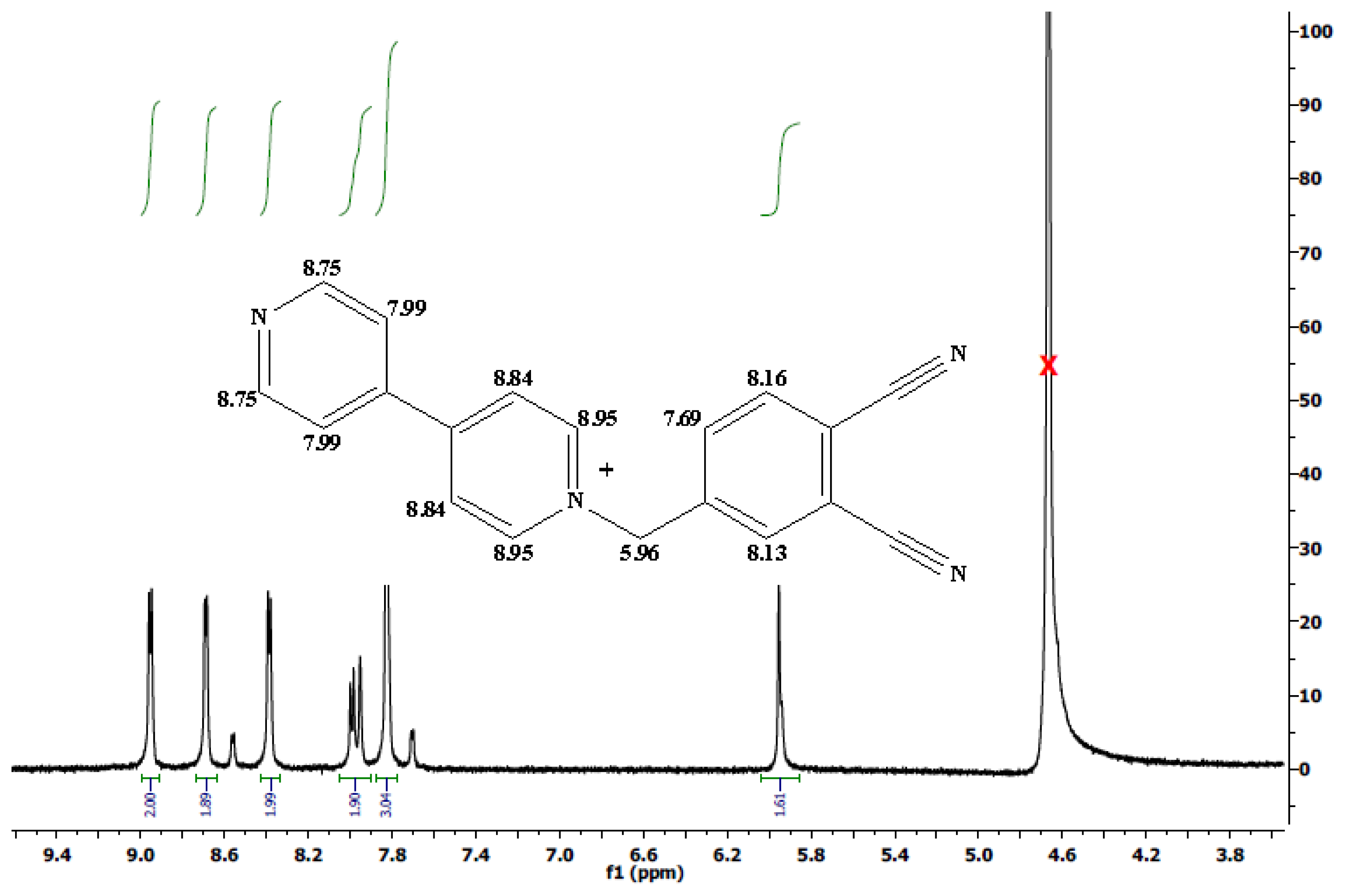

Supplement: Figure S3 — 1H-NMR spectrum of 1-(3,4-dicyanobenzyl)-[4,4′-bipyridine]-1-ium (PN1) (in DMSO -d6). [file turkjchem-47-5-1149s3.tif]

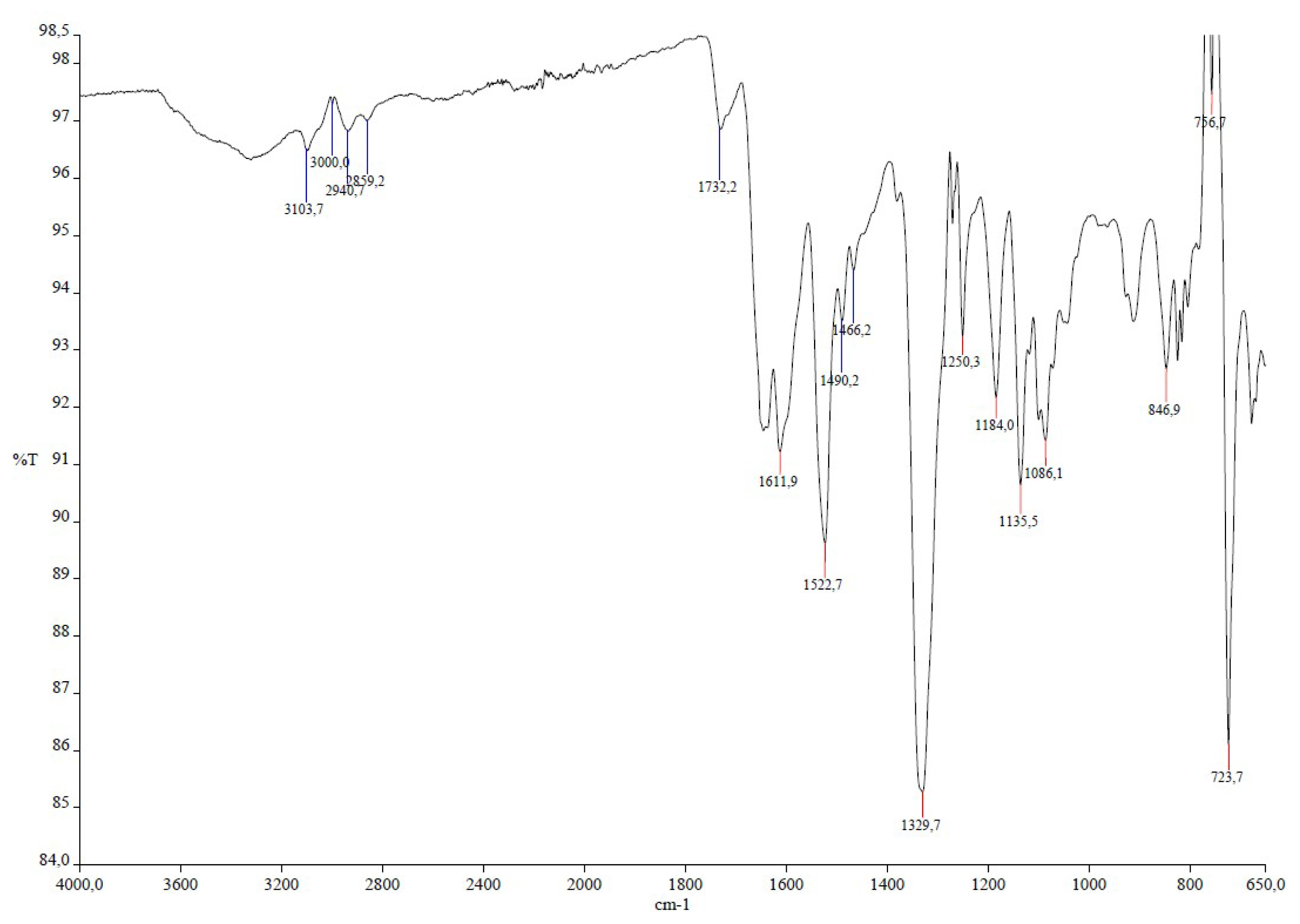

Supplement: Figure S4 — FT-IR spectrum of tetra-nitro phthalocyaninato zinc(II) (2). [file turkjchem-47-5-1149s4.tif]

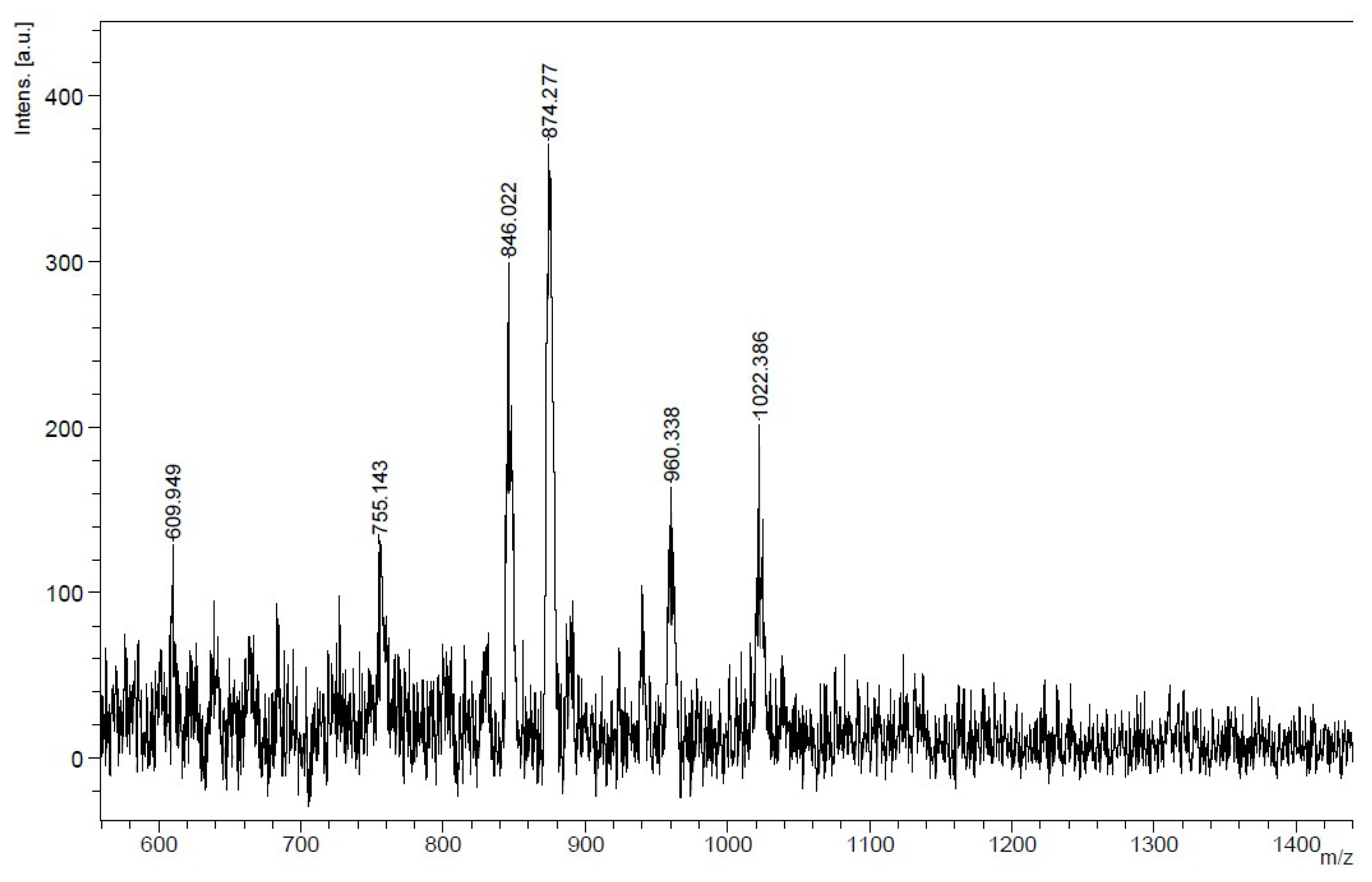

Supplement: Figure S5 — MS (MALDI-TOF) spectrum of tetra-nitro phthalocyaninato zinc(II) (2) (matrix: DHB). [file turkjchem-47-5-1149s5.tif]

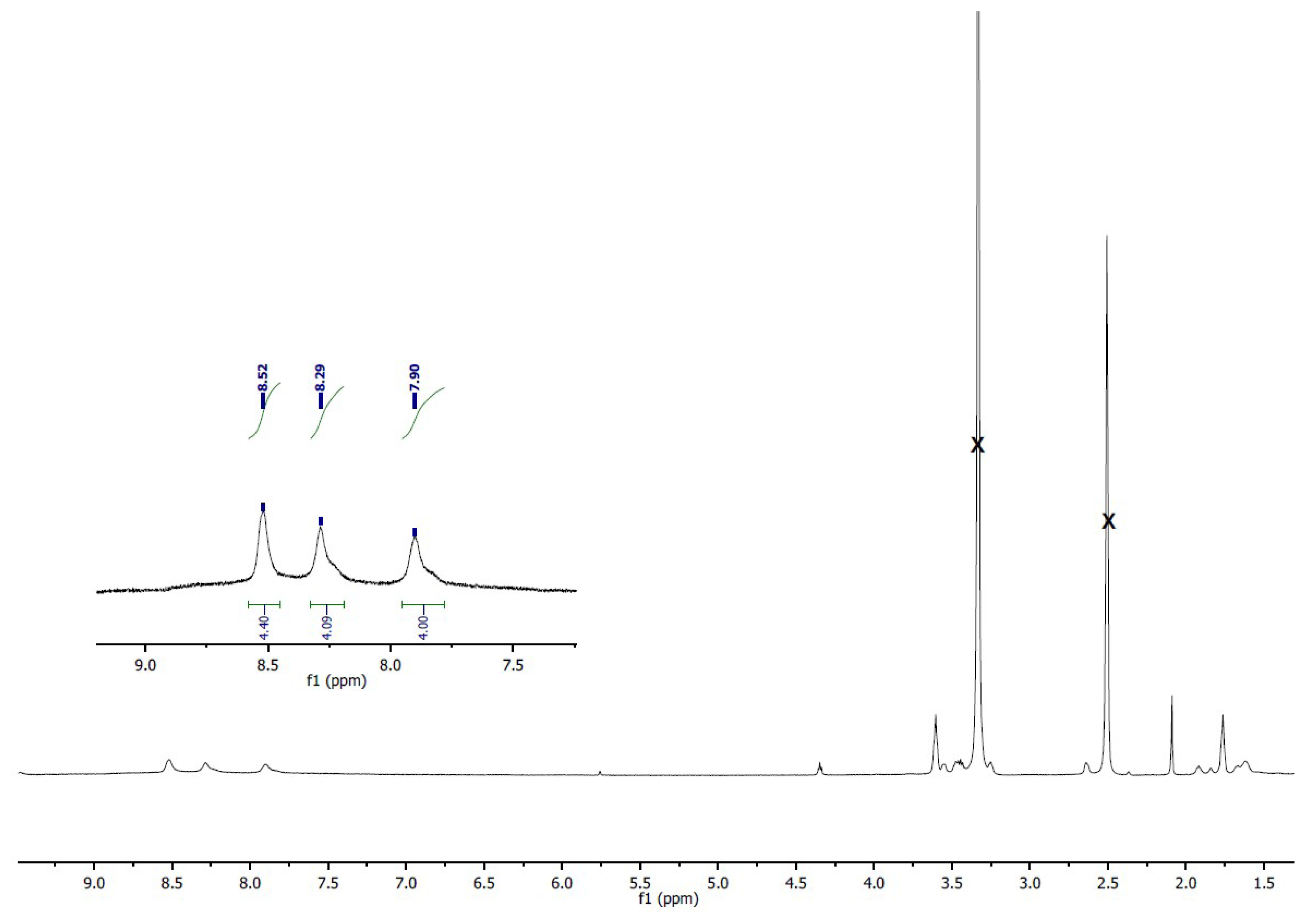

Supplement: Figure S6 — 1H-NMR spectrum of tetra-nitro phthalocyaninato zinc(II) (2) (in DMSO -d6). [file turkjchem-47-5-1149s6.tif]

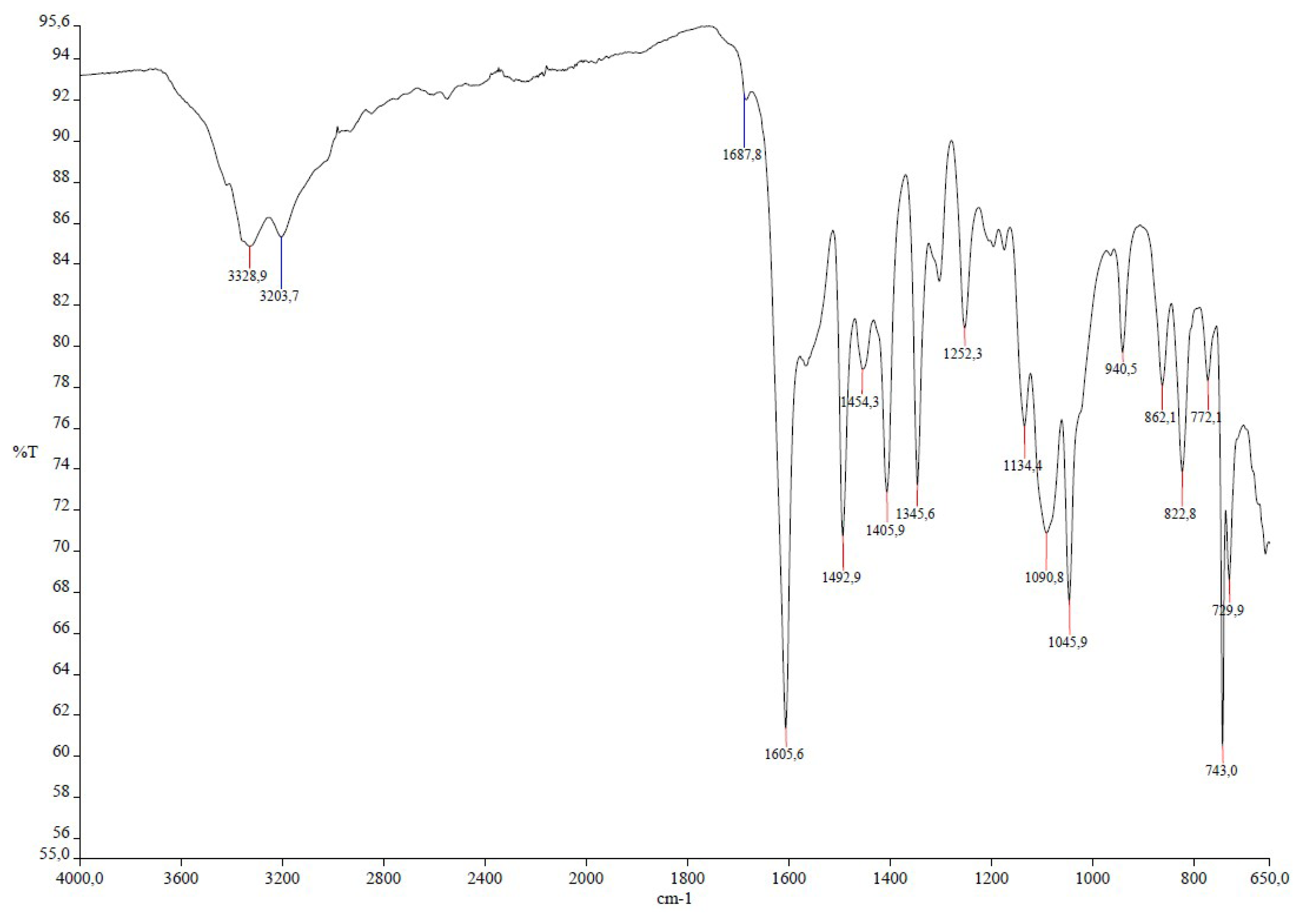

Supplement: Figure S7 — FT-IR spectrum of tetra-amino phthalocyaninato zinc(II) (3). [file turkjchem-47-5-1149s7.tif]

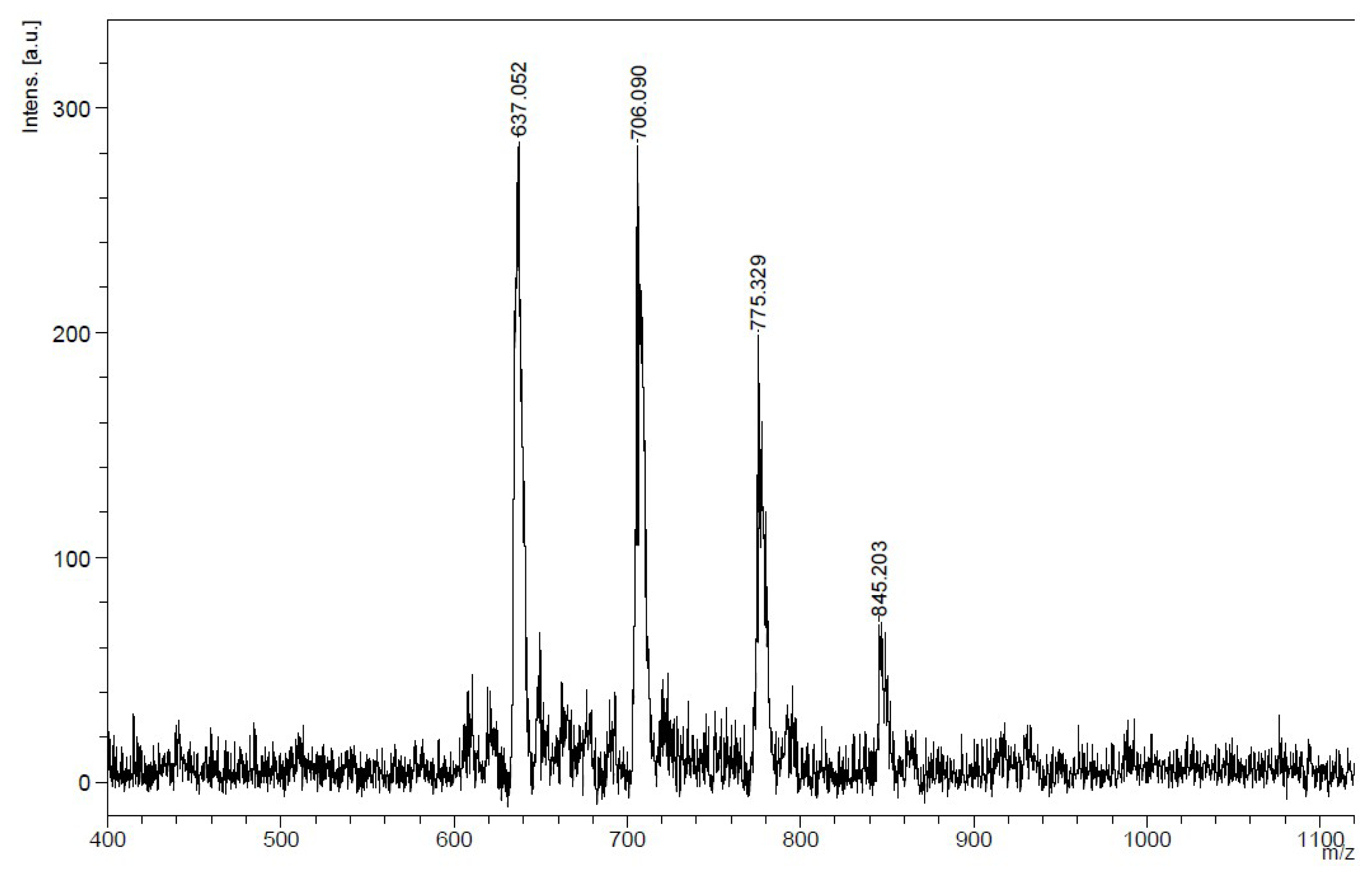

Supplement: Figure S8 — MS (MALDI-TOF) spectrum of tetra-amino phthalocyaninato zinc(II) (3) (matrix: DHB). [file turkjchem-47-5-1149s8.tif]

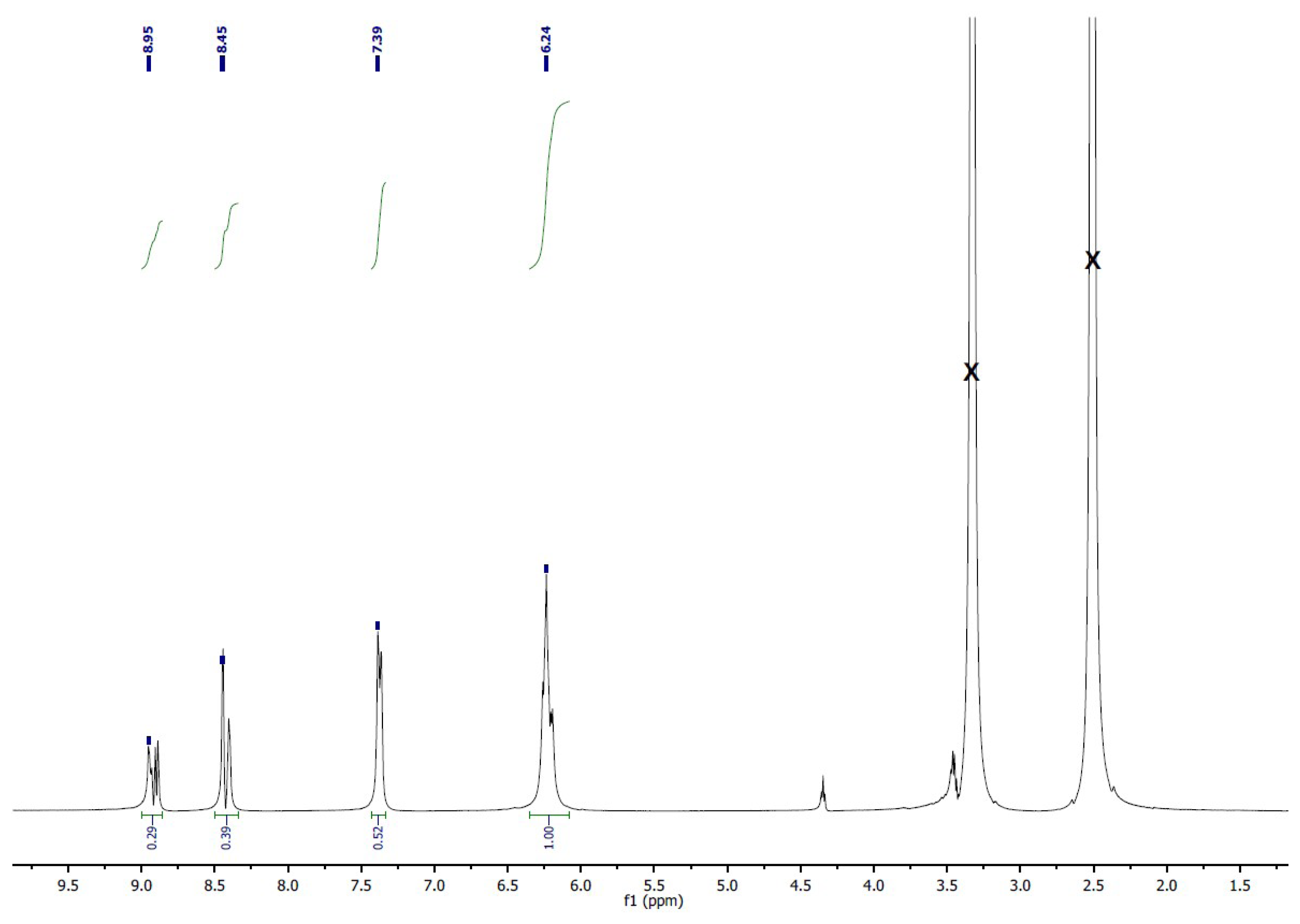

Supplement: Figure S9 — 1H-NMR spectrum of tetraamino phthalocyaninato zinc(II) (3) (in DMSO -d6). [file turkjchem-47-5-1149s9.tif]

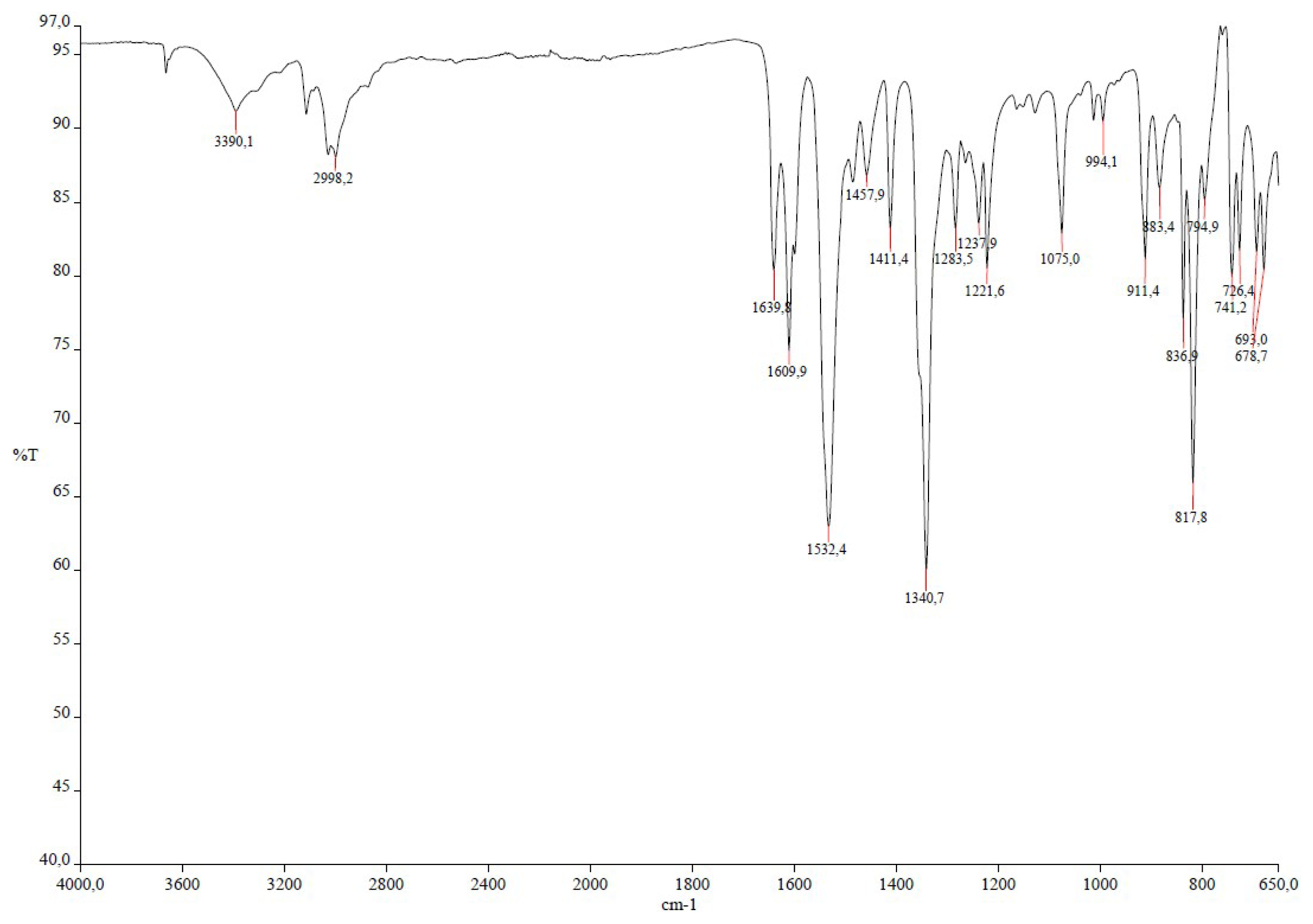

Supplement: Figure S10 — FT-IR spectrum of N-(2,4-dinitrophenyl)-4,4-bipyridinium chloride (1). [file turkjchem-47-5-1149s10.tif]

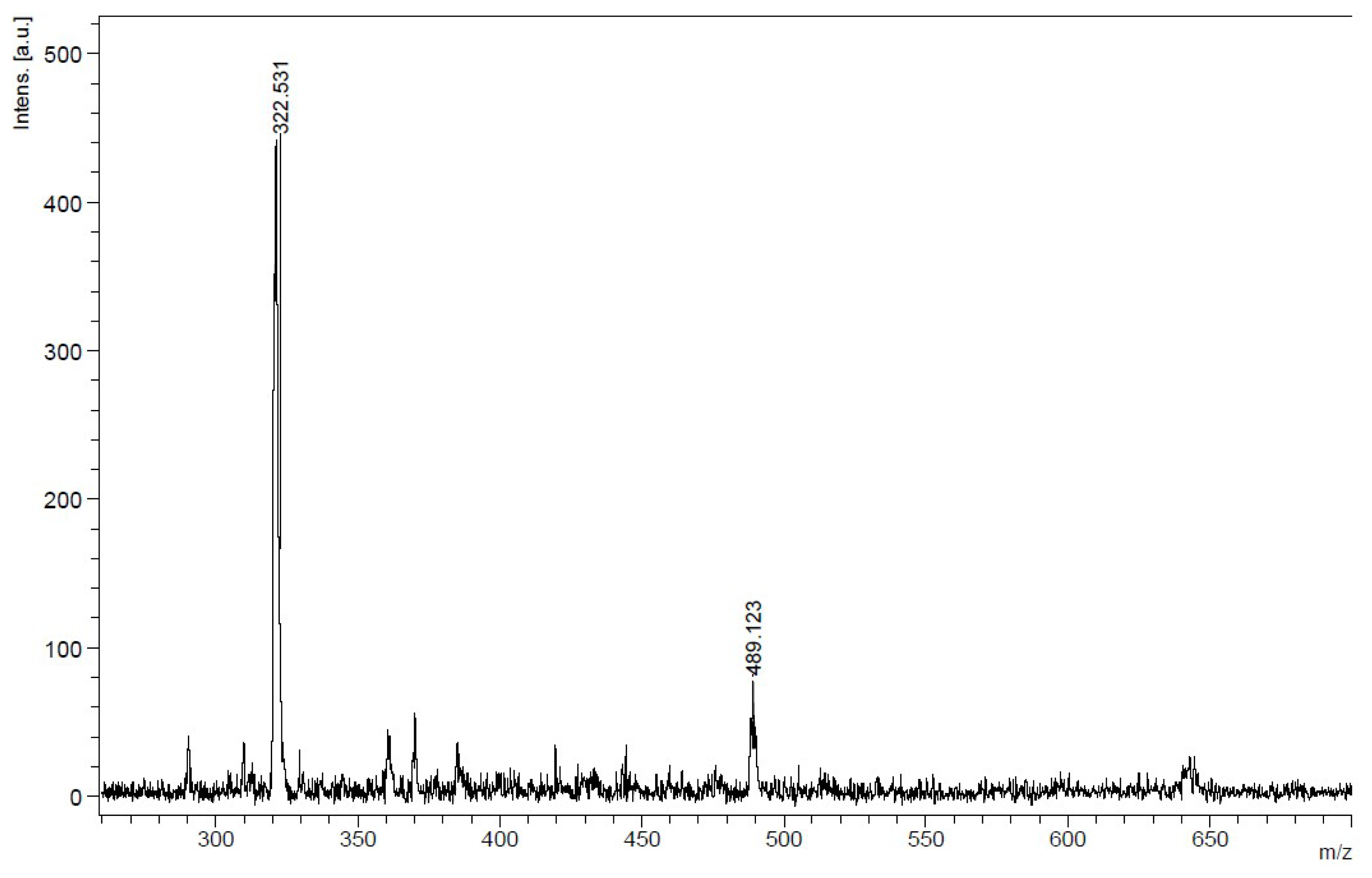

Supplement: Figure S11 — MS (MALDI-TOF) spectrum of N-(2,4-Dinitrophenyl)-4,4-bipyridinium chloride (1). [file turkjchem-47-5-1149s11.tif]

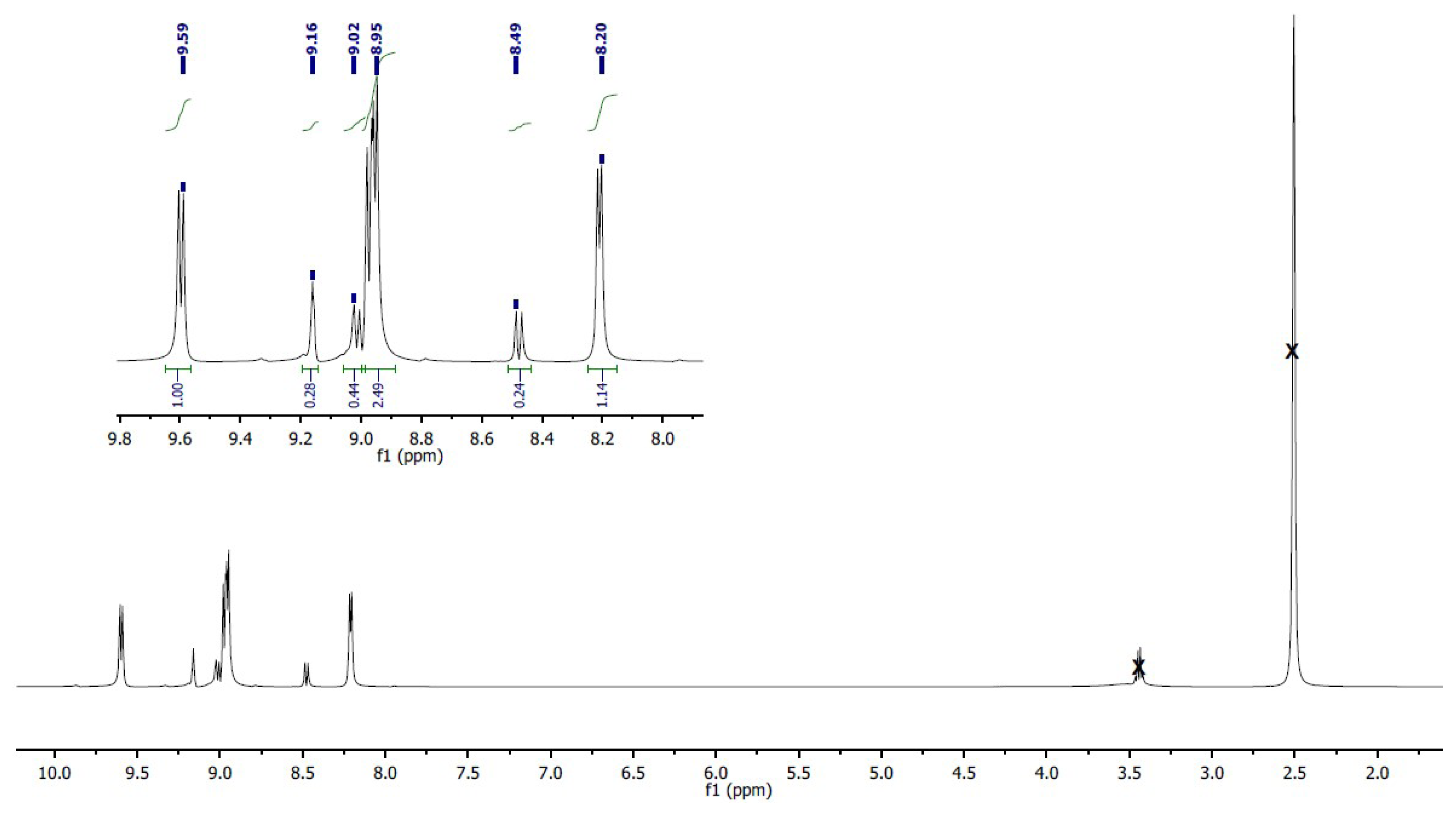

Supplement: Figure S12 — 1H-NMR spectrum of N-(2,4-Dinitrophenyl)-4,4-bipyridinium chloride (1) (in DMSO -d6). [file turkjchem-47-5-1149s12.tif]

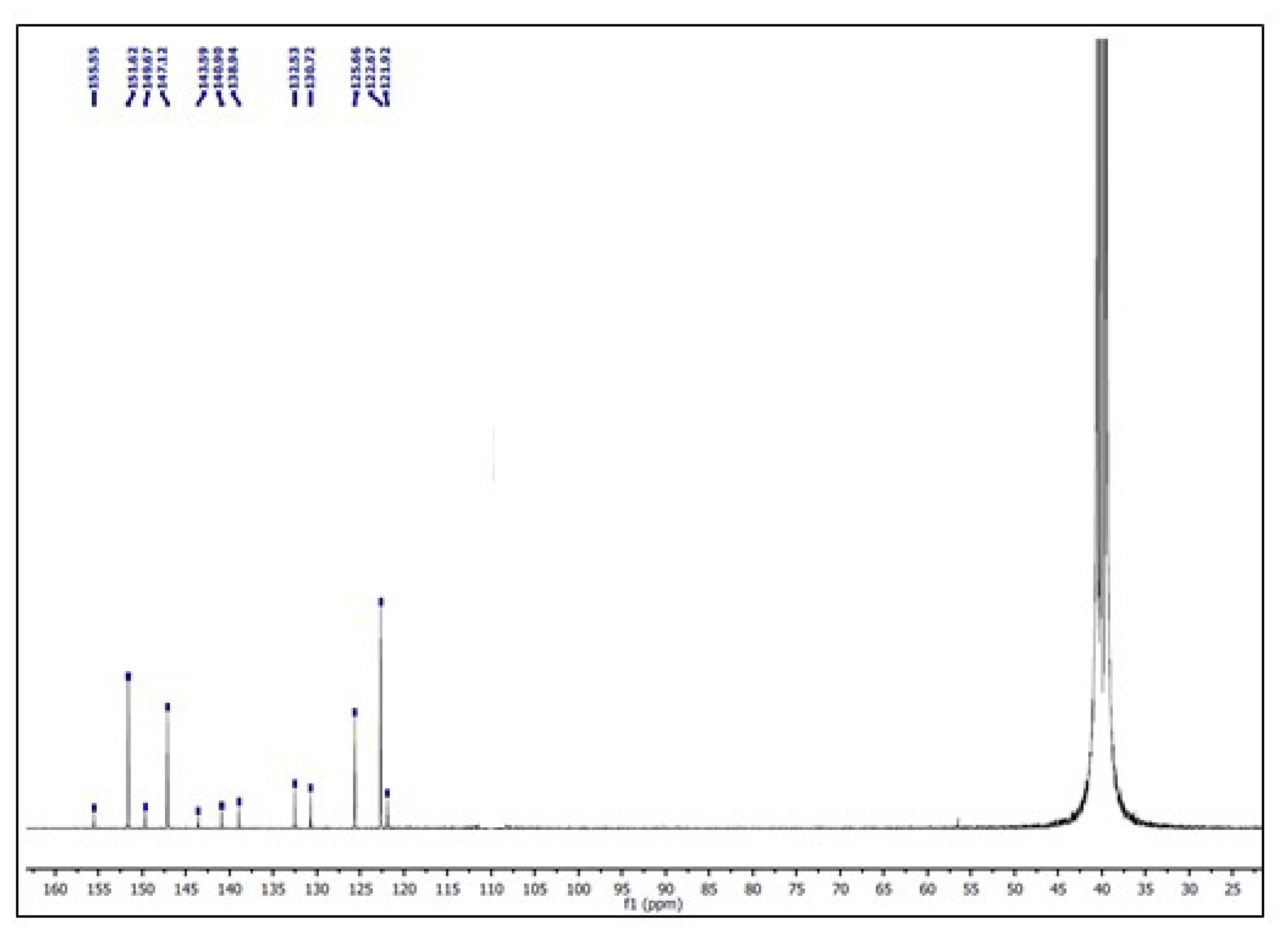

Supplement: Figure S13 — 13C-NMR spectrum of N-(2,4-Dinitrophenyl)-4,4-bipyridinium chloride (1) (in DMSO -d6). [file turkjchem-47-5-1149s13.tif]

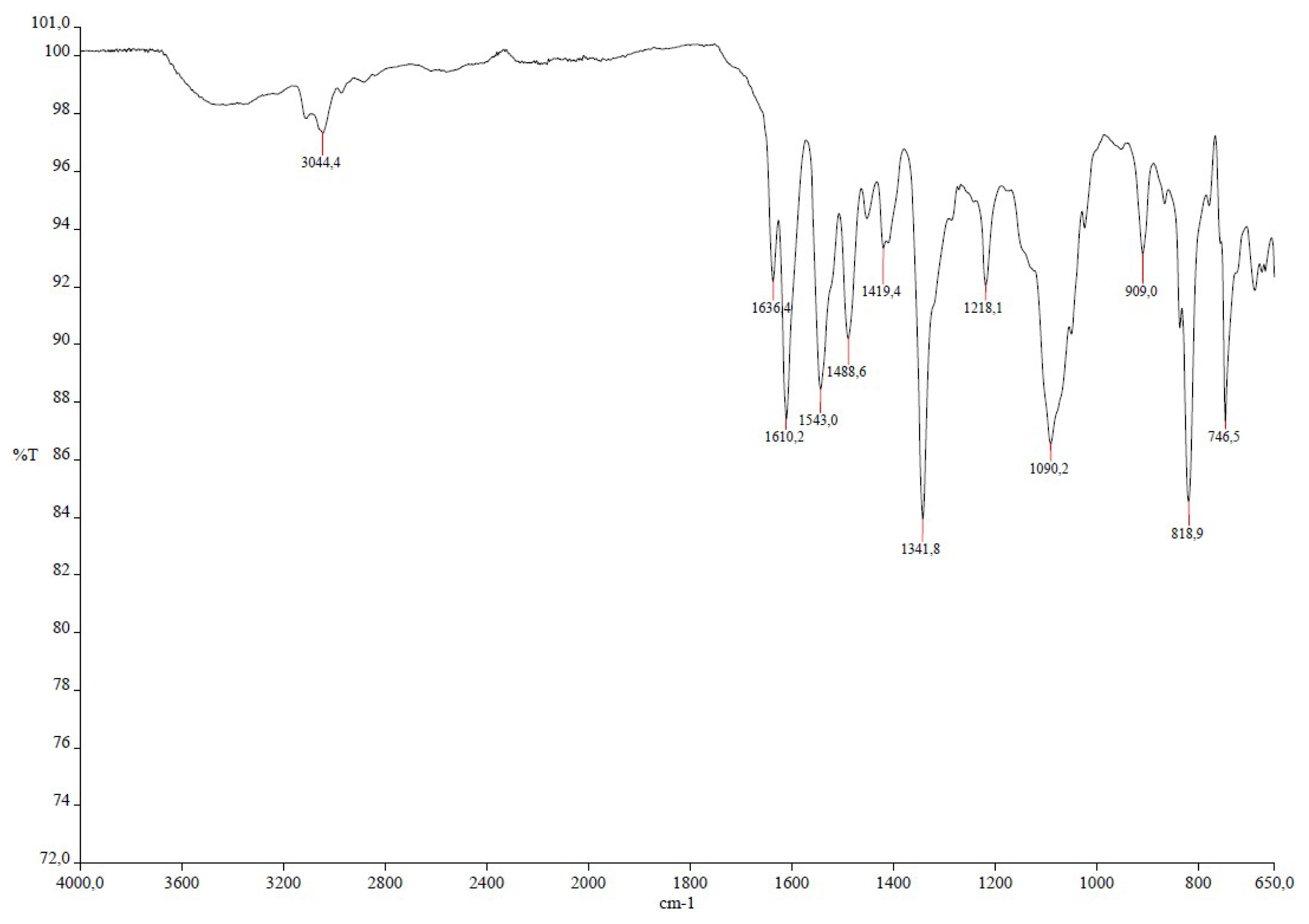

Supplement: Figure S14 — FT-IR spectrum of compound PcV1. [file turkjchem-47-5-1149s14.tif]

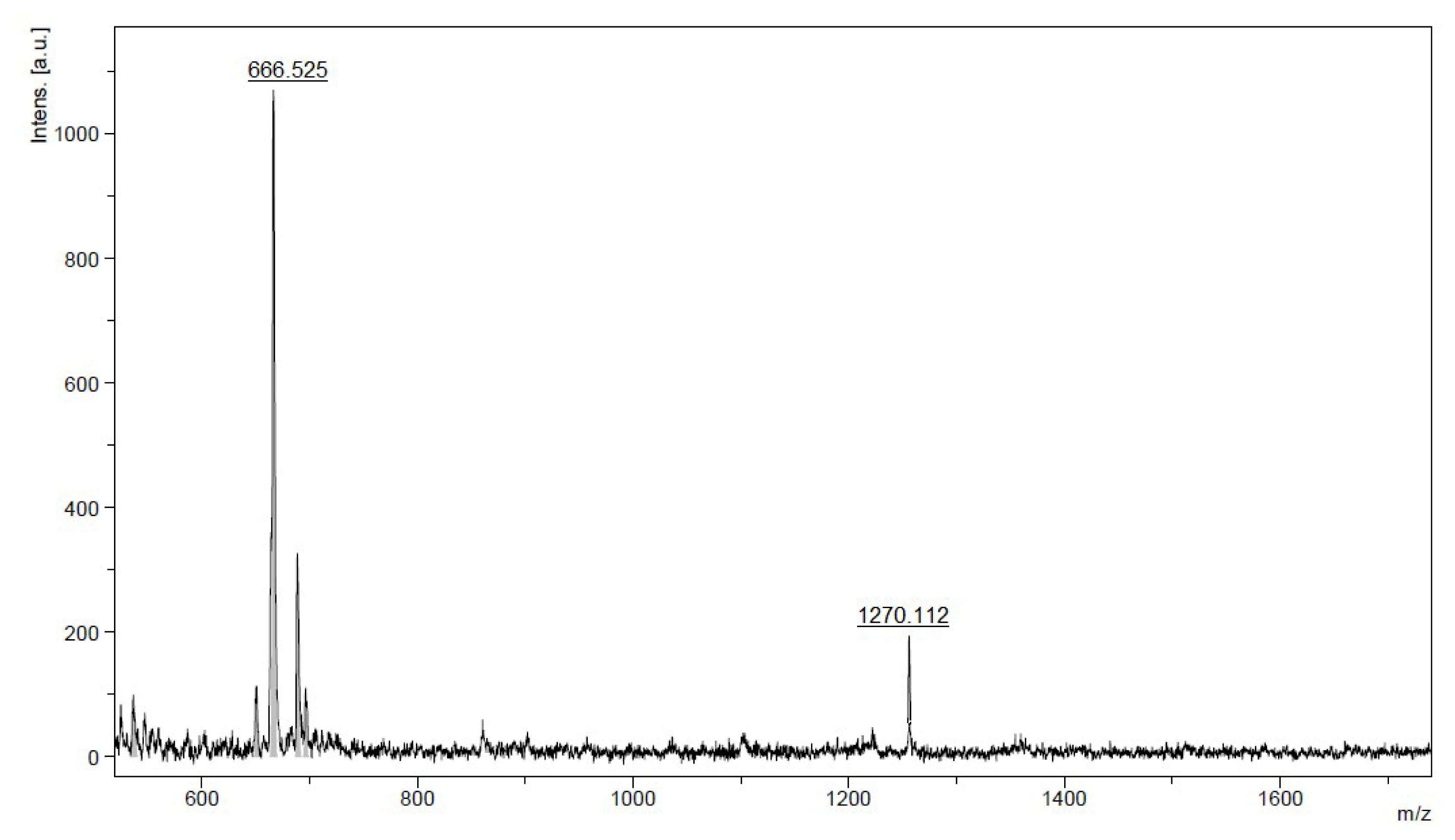

Supplement: Figure S15 — MS (MALDI-TOF) spectrum of compound PcV1. [file turkjchem-47-5-1149s15.tif]

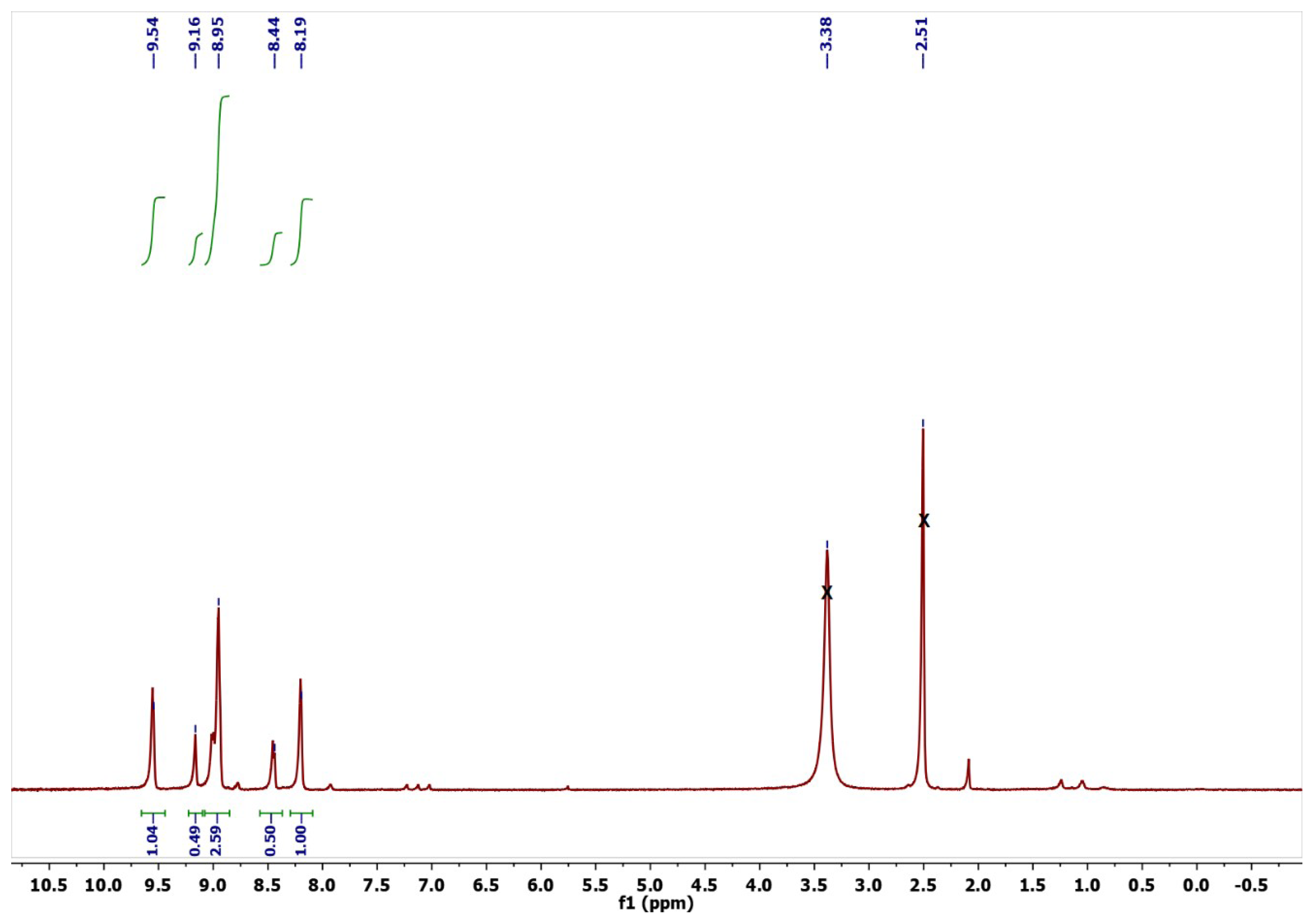

Supplement: Figure S16 — 1H-NMR spectrum of compound PcV1 (in DMSO -d [file turkjchem-47-5-1149s16.tif]

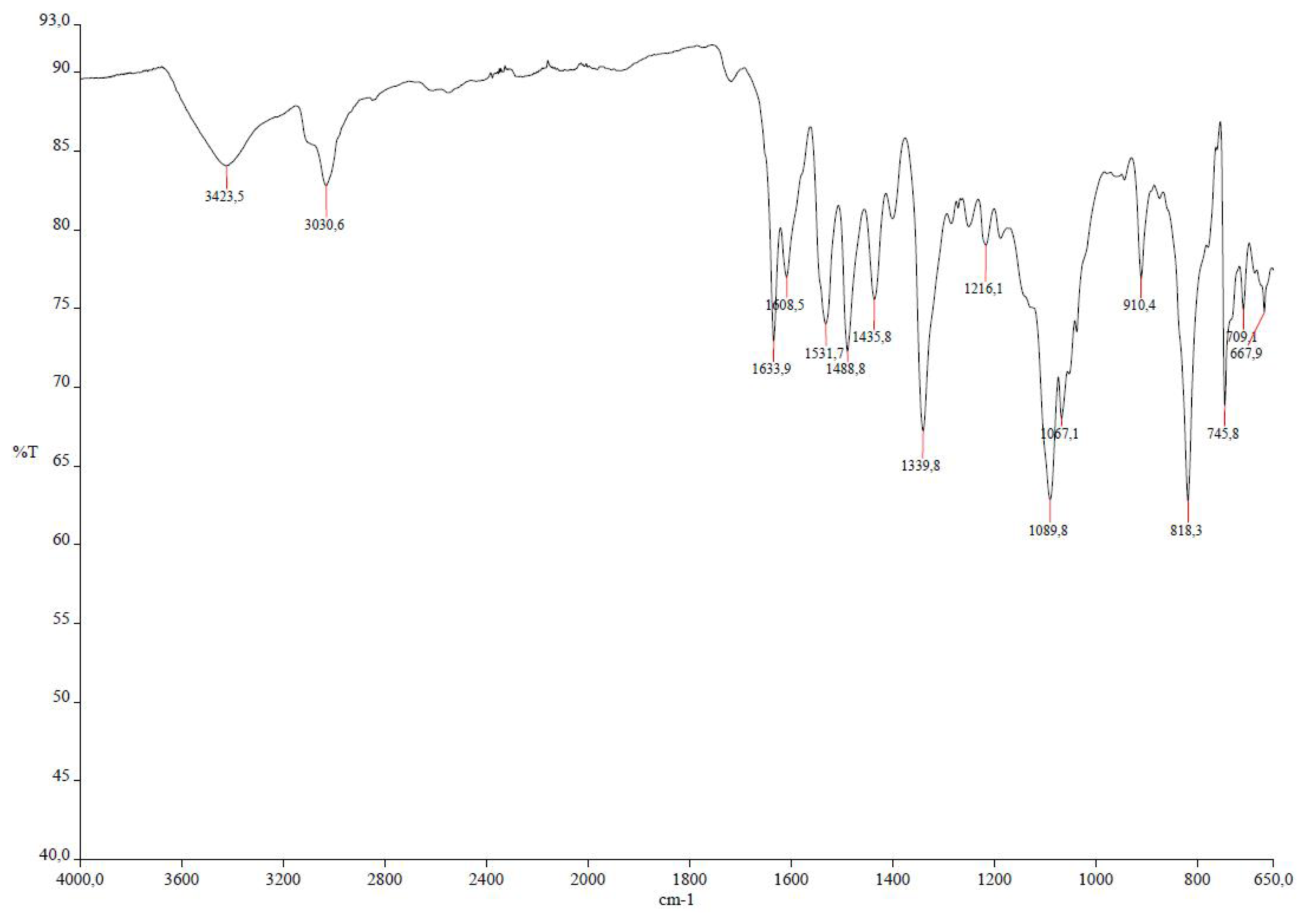

Supplement: Figure S17 — FT-IR spectrum of compound PcV2. [file turkjchem-47-5-1149s17.tif]

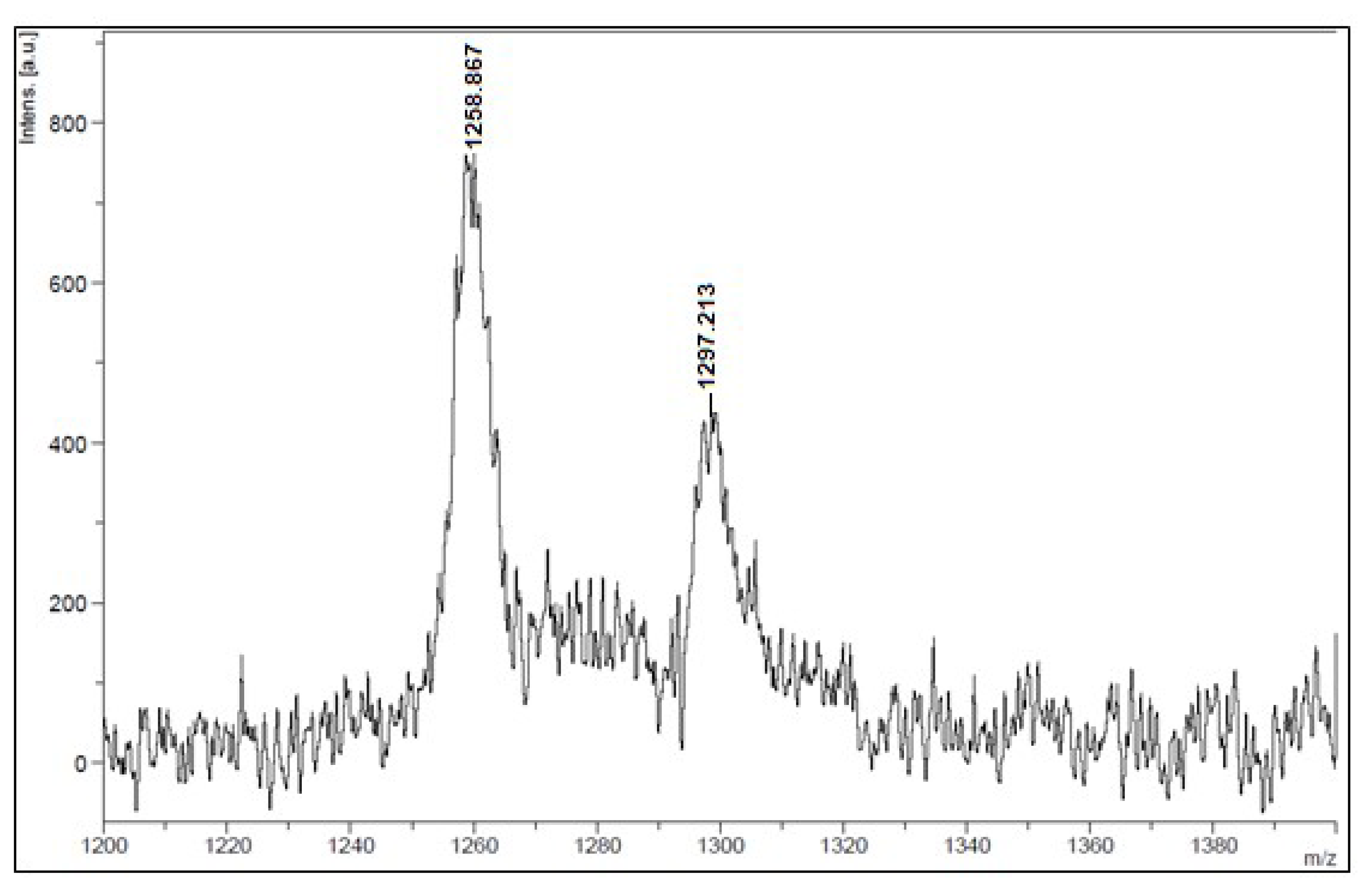

Supplement: Figure S18 — MS (MALDI-TOF) spectrum of compound PcV2. [file turkjchem-47-5-1149s18.tif]

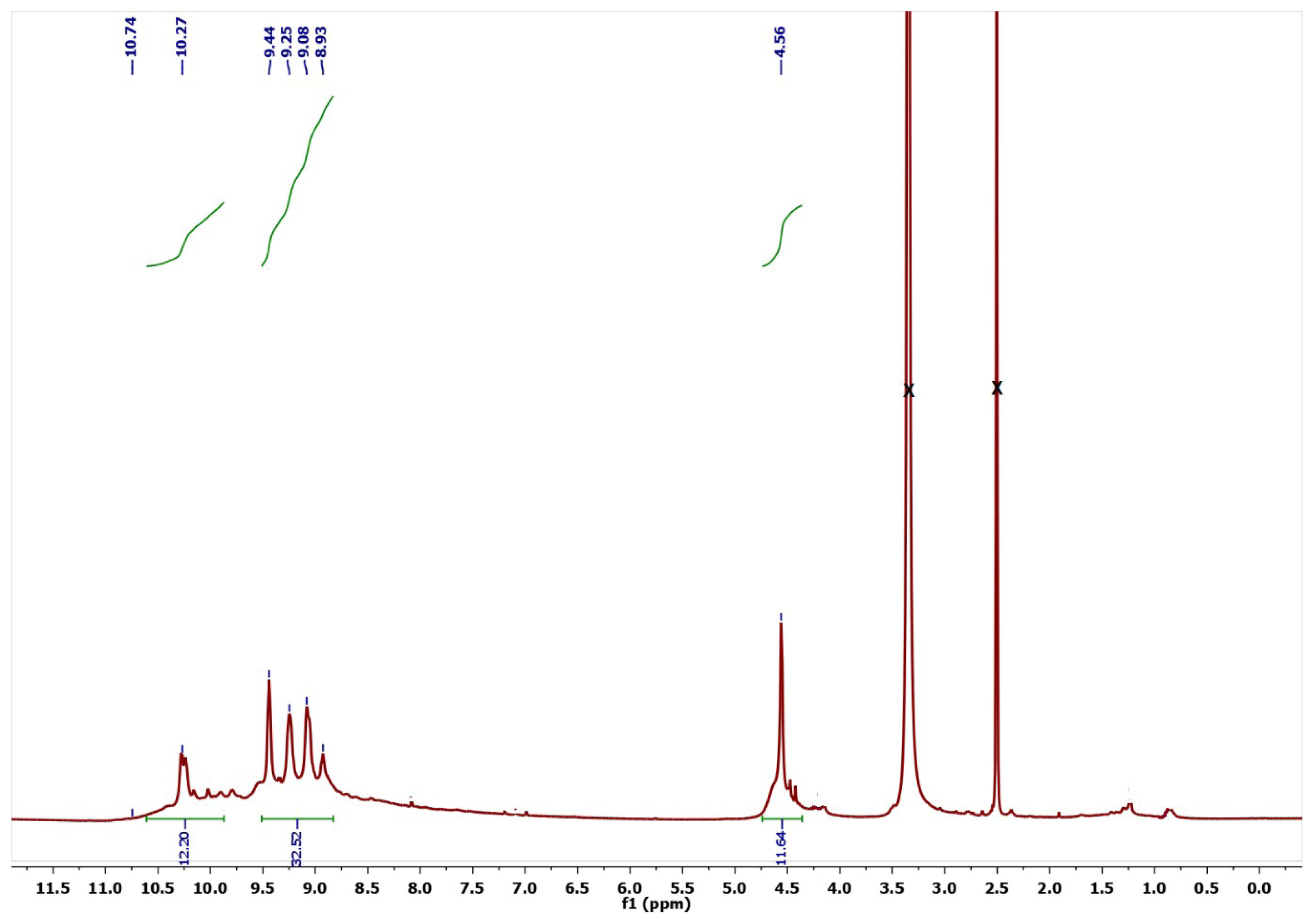

Supplement: Figure S19 — 1H-NMR spectrum of compound PcV2 (in DMSO -d6) [file turkjchem-47-5-1149s19.tif]

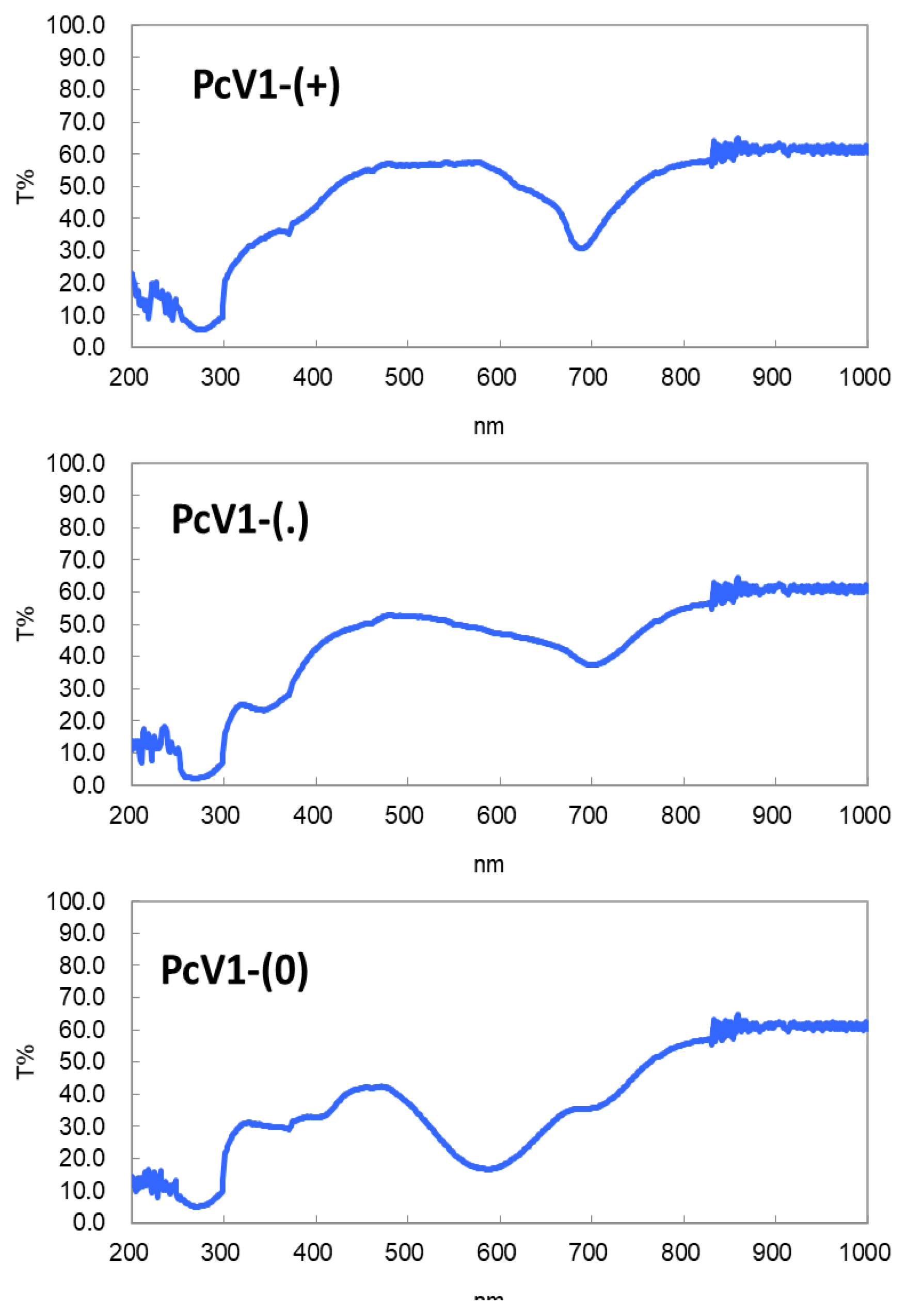

Supplement: Figure S17 — Transmittance spectra of PcV1-(+), PcV1-(.) and PcV1-(0) in DMSO (1 × 10-−5 M). [file turkjchem-47-5-1149s20.tif]

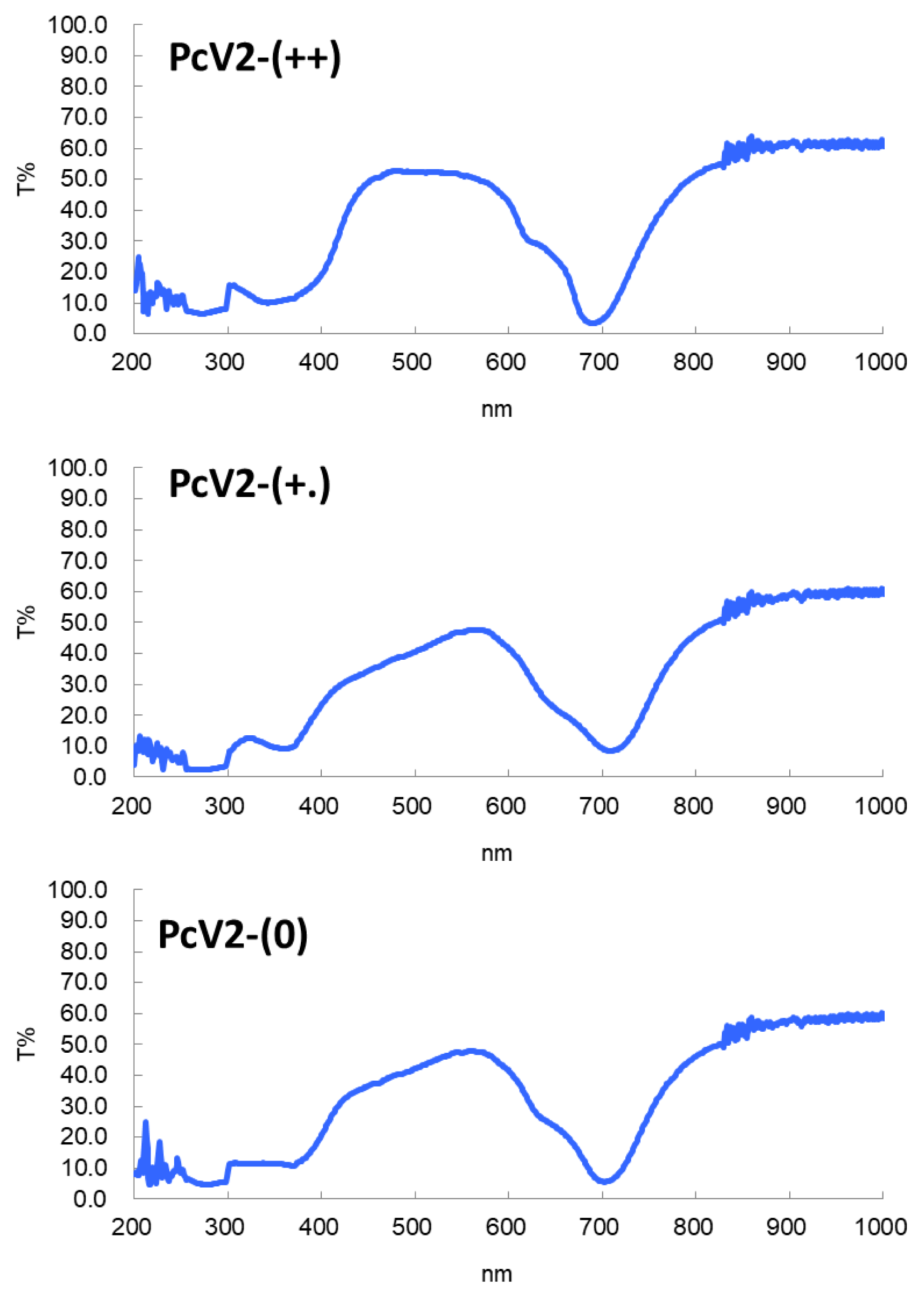

Supplement: Figure S18 — Transmittance spectra of PcV2-(++), PcV2-(+.) and PcV2-(0) in DMSO (1 × 10-−5 M). [file turkjchem-47-5-1149s21.tif]

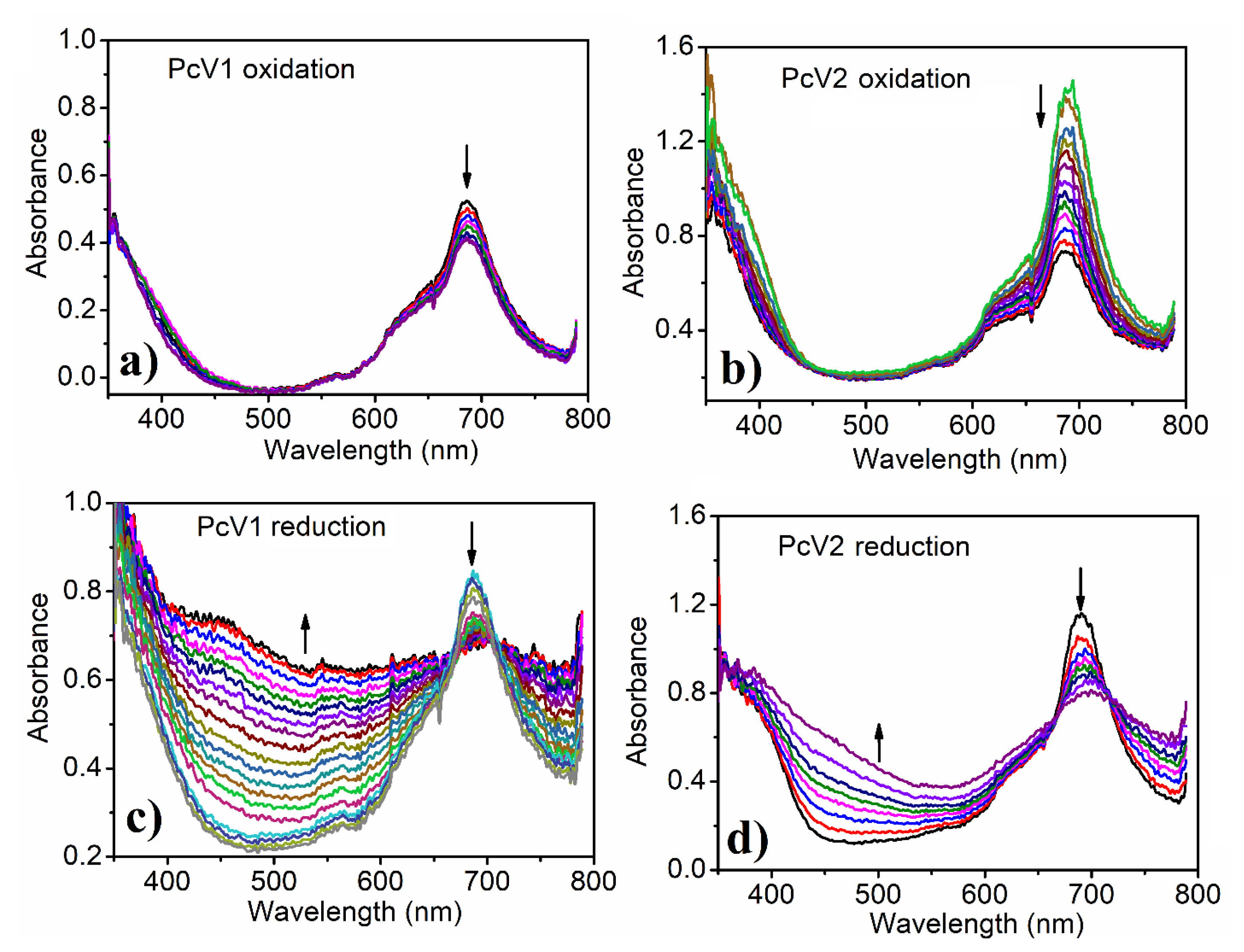

Supplement: Figure S19 — In situ UV-vis spectral changes during the electrolysis of PcV1 (a and c) and PcV2 (b and d) at various constant potentials in DMSO /TBAP electrolyte system. [file turkjchem-47-5-1149s22.tif]
